# Diagnostic agreement between Rhinoswabs and joint nose and throat swabs for the detection of influenza and SARS-CoV-2 among symptomatic ambulatory patients in Hong Kong

**DOI:** 10.1371/journal.pone.0355869

**Published:** 2026-08-11

**Authors:** Caitriona Murphy, Samuel M. S. Cheng, Loretta Mak, Hau Chi So, Dennis K. M. Ip, Malik Peiris, Benjamin J. Cowling

**Affiliations:** 1 World Health Organization Collaborating Centre for Infectious Disease Epidemiology and Control, School of Public Health, The University of Hong Kong, Hong Kong Special Administrative Region, China; 2 Centre of Immunology and Infection, Hong Kong Science and Technology Park, Hong Kong Special Administrative Region, China; Hong Kong Adventist Hospital, CHINA

## Abstract

**Background:**

Accurate detection of respiratory viruses relies on appropriate sample collection, but whether joint nose and throat sampling is necessary for high diagnostic performance or whether nose sampling alone can provide comparable results remains uncertain.

**Objectives:**

We evaluated the diagnostic agreement between joint nose and throat swabs (NTS) and Rhinoswab (RhinoMed, Cremorne, Australia), a novel anterior nasal swab for RT-qPCR detection of respiratory viruses among symptomatic outpatients.

**Study design:**

Outpatients with febrile acute respiratory illness were enrolled at a clinic in Hong Kong and paired joint NTS and Rhinoswab samples were tested by reverse transcription polymerase chain reaction (RT-qPCR) for influenza A, influenza B, SARS-CoV-2 and human ribonuclease P (RNase P) as an internal control. Diagnostic agreement and RT-qPCR Ct values were compared between the paired samples.

**Results:**

Between May 2023 and April 2024, 488 participants provided paired samples. Overall concordance between Rhinoswab and NTS was 98.0% (95% CI: 96.3% to 99.0%) for influenza A, 99.0% (95% CI: 97.6% to 99.7%) for influenza B, and 99.0% (95% CI: 97.6% to 99.7%) for SARS-CoV-2. Cohen’s kappa also indicated high agreement for all viruses: 0.95 (95% CI: 0.92 to 0.98) for influenza A, 0.94 (95% CI: 0.88 to 0.99) for influenza B, and 0.92 (95% CI: 0.85 to 0.99) for SARS-CoV-2. Ct values from Rhinoswab were moderately correlated with NTS for all three viruses and RNase P. The majority of discordant samples had Ct values of 30 and above (16/18, 88.9%).

**Conclusions:**

Rhinoswabs achieved high agreement with joint NTS for RT-qPCR detection of influenza and SARS-CoV-2. Given their minimal invasiveness Rhinoswabs are a viable alternative specimen type for supervised respiratory virus testing.

## Background

Accurate and timely detection of influenza and SARS-CoV-2 is important for surveillance and clinical management. Molecular testing by polymerase chain reaction (PCR) is the gold standard but its accuracy is contingent on the quality of the specimen collected which is influenced by the anatomical site of active viral replication sampled. In Hong Kong, diagnostic testing is typically carried out using nasopharyngeal swabs or joint nose and throat swabs (NTS). In contrast to nasopharyngeal swabbing, which requires deep insertion into the nasal cavity by a trained professional, joint NTS is less invasive and suitable for self-collection. However, an evaluation of the UK COVID-19 National Testing Programme observed a preference for nose-only swabbing compared to joint NTS, reporting it was easier to perform and less uncomfortable [1]. The Rhinoswab™ was developed by RhinoMed (Cremorne, Australia) in 2020 [2,3] aiming to be minimally invasive by collecting nasal discharge from the anterior nares.

## Objectives

We aimed to estimate the concordance between Rhinoswabs and joint NTS for detecting influenza and SARS-CoV-2 via RT-PCR among symptomatic outpatients in Hong Kong.

## Study design

### Study participants

We utilised data from an ongoing outpatient surveillance study in Hong Kong, between 03/05/2023 and 29/04/2024. Eligible patients were aged ≥6 months and were seeking medical care for a febrile acute respiratory illness, defined as the presence of ≥2 respiratory symptoms within 3 days of symptom onset. A questionnaire was administered to collect vaccination history and demographics. The study protocol was approved by the Institutional Review Board of the University of Hong Kong. Written informed consent was obtained from all participants or their legal guardians for minors. The individual pictured in this manuscript has given written informed consent to publish the image.

### Specimen collection and laboratory testing

All participants were first sampled using rapid antigen tests (unrelated to this study), followed by a joint NTS (CLASSIQSwabs™, Copan) and then by the Rhinoswab as participants could opt out of being sampled using a Rhinoswab. A demonstration video was shown and a junior version of the Rhinoswab was available for children (S1 Fig). For participants that agreed to provide a Rhinoswab sample, collection was performed by study staff according to manufacturers’ instructions. Swabs were inserted into the anterior nares and left for 15 seconds while the participant breathed normally and then moved back and forth in the nostril for an additional 15 seconds. After removal, each loop of the swab was snapped off into the collection tube at predefined break points. All swabs were transported to the laboratory in viral transport media and tested for influenza A, influenza B, and SARS-CoV-2 using a ViiA7 RT-qPCR system (ThermoFisher). RNA extraction and RT-qPCR targets and conditions have been described previously [4]. A cycle threshold (Ct) value of <40 was considered positive.

### Statistical analysis

Participants were included if they had paired joint NTS and Rhinoswab samples. Agreement was assessed by estimating concordance, defined as the percentage of paired swabs with identical results (positive or negative) and Cohen’s kappa with 95% confidence intervals (CI). Concordance was also evaluated by age, time since the onset of symptoms and vaccination status (received influenza or COVID-19 vaccination within a year before seeking medical care). Sensitivity, specificity, positive predictive value (PPV), and negative predictive value (NPV) with 95% confidence intervals are also reported to evaluate the diagnostic performance of the Rhinoswab compared to joint NTS.

In addition, the correlation between the Ct values for both swabs was assessed using Pearson’s correlation coefficients. We plotted the difference in Ct values (Rhinoswab minus NTS) against NTS Ct values to assess variability across a range of viral loads. Analyses were performed using R version 4.0.2 (R Foundation for Statistical Computing, Vienna, Austria).

## Results

During the study period, 531 participants were enrolled and invited to provide paired samples, of whom 488 (91.9%) provided paired samples, while 43 (8.1%) opted out. Of those that opted out, 16/43 (37.2%) were unwilling after viewing the demonstration video. Of the 488 Rhinoswabs collected, 246 (50.4%) were Rhinoswab junior. The mean age was 25 years and the majority of participants were female (55.5%) (Table 1).

**Table 1:** Study participant characteristics by virus detected by both Rhinoswab and joint nose-throat swab.

Characteristic	Influenza A (n = 136)	Influenza B (n = 42)	SARS-CoV-2 (n = 32)	Negative (n = 260)	Discordant (n = 18)	Overall (n = 488)
Age, Mean (SD)	25.6 (20.3)	25.1 (15.9)	41.7 (22.1)	22.4 (20.3)	29.2 (18.5)	25.1 (20.5)
Male	62 (45.6%)	23 (54.8%)	10 (31.3%)	117 (45.0%)	5 (27.8%)	217 (44.5%)
Chronic condition	12 (8.8%)	2 (4.8%)	5 (15.6%)	28 (10.8%)	3 (16.7%)	50 (10.2%)
**Vaccinated within a year**						
Influenza vaccine	41 (30.1%)	5 (11.9%)	9 (28.1%)	111 (42.7%)	5 (27.8%)	171 (35.0%)
COVID-19 vaccine	6 (4.4%)	0 (0%)	1 (3.1%)	4 (1.5%)	1 (5.6%)	12 (2.5%)
**Symptom onset**						
≤ 24 hours	71 (52.2%)	12 (28.6%)	26 (81.3%)	132 (50.8%)	12 (66.7%)	253 (51.8%)
> 24–47 hours	27 (19.9%)	8 (19.0%)	3 (9.4%)	38 (14.6%)	3 (16.7%)	79 (16.2%)
> 48–72 hours	38 (27.9%)	22 (52.4%)	3 (9.4%)	90 (34.6%)	3 (16.7%)	156 (32.0%)

Influenza A was detected in 136 participants by both joint NTS and Rhinoswab, with a concordance of 98.0% (95% CI: 96.3% to 99.0%) and Cohen’s kappa of 0.95 (95% CI: 0.92 to 0.98). There were 42 influenza B and 32 SARS-CoV-2 positives by both swabs respectively (Table 1). The concordance for detecting influenza B and SARS-CoV-2 was 99.0% (95% CI: 97.6% to 99.7%) for both viruses, with corresponding Cohen’s kappa values of 0.94 (95% CI: 0.88 to 0.99) and 0.92 (95% CI: 0.85 to 0.99), respectively. Concordance was similar among different age groups, time since onset and vaccination status (S2 Fig).

**Fig 1:**
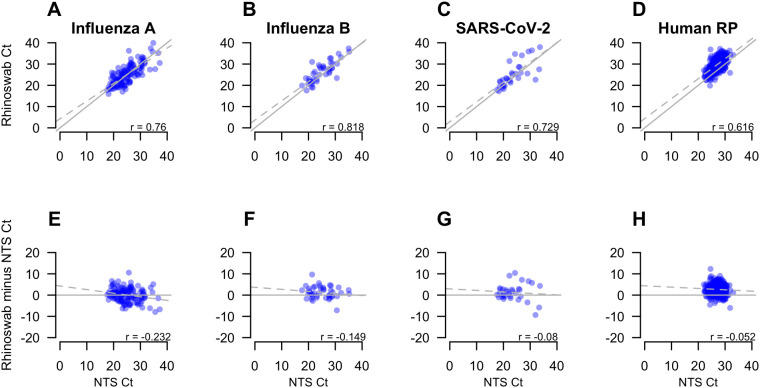
Correlation of cycle threshold (Ct) values between joint NTS and Rhinoswab for influenza A, influenza B, SARS-CoV-2 test positives and the internal control, human ribonuclease P (panels A–D). The bottom row (panels E–H) shows the difference in Ct-values (Rhinoswab Ct minus NTS Ct) plotted against the NTS Ct-values. Solid lines represent a perfect agreement, and dotted lines indicate the fitted linear regression. Pearson correlation coefficients (r) are displayed in each panel.

The Ct values from Rhinoswabs were significantly positively correlated with those from joint NTS for all three viruses and the internal control, human RNase P (Fig 1 panels A-D). Correlations coefficients were 0.76 for influenza A, 0.818 for influenza B, and 0.729 for SARS-CoV-2, while human RNase P showed a moderate correlation (r = 0.616). When the difference between Ct values (Rhinoswab minus NTS) was plotted against the NTS Ct value there was no evidence of substantially different Rhinoswab agreement for higher or lower Ct values (Fig 1 panels E-H). The majority of discordant samples (16/18, 88.9%) had Ct values of ≥30 (S1 Table) and occurred within 24 hours of symptom onset (Table 1).

When comparing the Rhinoswab to joint NTS as the reference test, sensitivity was 95.8% (95% CI: 91.0% to 98.4%) for influenza A, 91.3% (95% CI: 79.2% to 97.6%) for influenza B, and 91.4% (95% CI: 76.9% to 98.2%) for SARS-CoV-2. Specificity was 98.8% (95% CI: 97.1% to 99.7%) for influenza A, 99.8% (95% CI: 98.7% to 100.0%) for influenza B, and 99.6% (95% CI: 98.4% to 99.9%) for SARS-CoV-2. PPV ranged from 94.1% to 97.7%, while NPV were all above 98.3% (S2 Table).

## Discussion

Rhinoswabs had a high concordance with joint NTS for detecting influenza A (98.0%), influenza B (99.0%), and SARS-CoV-2 (99.0%). This is higher than the 95.2% concordance reported for SARS-CoV-2 estimated among symptomatic patients in Australia [2]. Two other studies have evaluated Rhinoswabs, observing a positive percent agreement of 96.2% for detecting six viruses in a hospital setting [3] and sensitivity of 95% in patients with high viral loads (Ct < 20) compared to combined oro-nasopharyngeal swabs in an emergency department population [5].

This is similar to the sensitivity for detecting influenza A and a bit higher than that for influenza B and SARS_CoV-2. The observed high concordance could be driven by a high proportion of concordant negative samples. Influenza B and SARS-CoV-2 had a lower prevalence resulting in a higher concordance (99.0%) due to a greater number of true negatives while influenza A had the highest prevalence in this study with a slightly lower concordance (98.0%). However, despite prevalence-driven differences, Cohen’s kappa remained consistently high across all viruses (0.92–0.95), indicating strong agreement between the two sampling methods beyond what would be expected by chance alone. Discordance was among samples with Ct values of ≥30, similar to previous literature for non-nasopharyngeal samples at low viral loads [6]. This may not affect case detection when transmissibility is greatest (higher viral loads) [7].

We observed a lower (compared to influenza and SARS-CoV-2) correlation for RNase P between Rhinoswab and joint NTS Ct values, which could reflect variability in the amount of cellular material collected by the two swab types. However, differences in host-cell yield do not necessarily translate into poorer viral RNA detection. For example, a comparison of oropharyngeal, nasopharyngeal and combined swabs observed varied RNase P and total human RNA levels but these differences did not correlate with viral load measured by RT-qPCR [8]. Similar patterns were reported for nasal and saliva specimens, suggesting more variable host-cell recovery while still maintaining comparable SARS-CoV-2 detection to nasopharyngeal swabs [9].

Rhinoswabs in this study were the third sequential sampling of the anterior nares, which may have reduced the amount of viral material available and lead to an underestimate of Rhinoswab agreement. Despite this conservative sampling sequence, Rhinoswabs maintained comparable concordance to joint NTS. This finding is similar to studies that carried out sequential sampling to compare self-collected samples followed by a healthcare worker collecting swabs, finding that self-collected nasal and mid-turbinate swabs yielded viral loads equivalent to or even higher than healthcare-worker-collected nasopharyngeal swabs [10,11]. Nevertheless, the fixed sampling order precludes assessment of whether agreement would be equivalent (or potentially superior) if the Rhinoswab had been collected first.

Limitations include sample collection early in the course of infection, when viral loads are typically highest. Consequently, the high concordance observed may be overestimated relative to patients with lower viral loads or asymptomatic infections. Rhinoswab collection was supervised by trained staff, and we did not evaluate self-collection. However, several studies have shown that self-collected nasal swabs yield viral loads and diagnostic performance similar to healthcare worker collected swabs [10,11]. Given the user-friendly design of Rhinoswabs the agreement could be similar between collection methods, but dedicated studies utilizing self-collected swabs and among asymptomatic are needed.

To conclude, supervised testing using Rhinoswabs demonstrated a high concordance with joint NTS for the detection of influenza and SARS-CoV-2 in symptomatic outpatients. Given Rhinoswabs only sample the nose and are child friendly, they could be a practical alternative for supervised respiratory virus testing in outpatient settings.

## Supporting information

S1 Fig Sample collection from outpatients using Rhinoswabs. Research staff collected samples using flocked nylon Rhinoswabs (available in adult and junior sizes). The swab design includes snap points to detach into a standard collection tube (Panel A). The sampling technique involves inserting the swab into both nostrils (Panel B). Collected samples were suspended in viral transport media and subsequently tested for influenza and SARS-CoV-2 via PCR.
(PDF)

S2 Fig Agreement of pooled NT swabs and Rhinoswabs by age, time since the onset of symptoms and vaccination status (vaccinated within a year). (PDF)

S1 Table Number of concordant and discordant results between Rhinoswab and joint NTS, with mean cycle threshold values for each category. (DOCX)

S2 Table Diagnostic performance of Rhinoswab compared to joint NTS by virus. (DOCX)
