# Supplementary material for: Diagnostic agreement between Rhinoswabs and joint nose and throat swabs for the detection of influenza and SARS-CoV-2 among symptomatic ambulatory patients in Hong Kong
Source: PLoS One. 2026 Aug 11;21(8):e0355869. doi: 10.1371/journal.pone.0355869 (PMC13460600; doi:10.1371/journal.pone.0355869)

Concordance among subgroups

Virus, subgroup	Total	Concordant	Estimate [95% CI]
**Influenza A**			
6 months to 17 years old	260	257	98.85 [96.67, 99.76]
18 to 64 years old	205	198	96.59 [93.09, 98.62]
65 years and above	27	27	100.00 [87.23, 100.00]
Vaccinated against influenza	174	170	97.70 [94.22, 99.37]
Unvaccinated against influenza	315	309	98.10 [95.90, 99.30]
Vaccinated against SARS−CoV−2	12	11	91.67 [61.52, 99.79]
Unvaccinated against SARS−CoV−2	99	97	97.98 [92.89, 99.75]
**Influenza B**			
6 months to 17 years old	260	258	99.23 [97.25, 99.91]
18 to 64 years old	205	202	98.54 [95.78, 99.70]
65 years and above	27	27	100.00 [87.23, 100.00]
Vaccinated against influenza	174	173	99.43 [96.84, 99.99]
Unvaccinated against influenza	315	312	99.05 [97.24, 99.80]
Vaccinated against SARS−CoV−2	12	12	100.00 [73.54, 100.00]
Unvaccinated against SARS−CoV−2	99	99	100.00 [96.34, 100.00]
**SARS−CoV−2**			
6 months to 17 years old	260	259	99.62 [97.88, 99.99]
18 to 64 years old	205	201	98.05 [95.08, 99.47]
65 years and above	27	27	100.00 [87.23, 100.00]
Vaccinated against influenza	174	173	99.43 [96.84, 99.99]
Unvaccinated against influenza	315	311	98.73 [96.78, 99.65]
Vaccinated against SARS−CoV−2	12	12	100.00 [73.54, 100.00]
Unvaccinated against SARS−CoV−2	99	98	98.99 [94.50, 99.97]

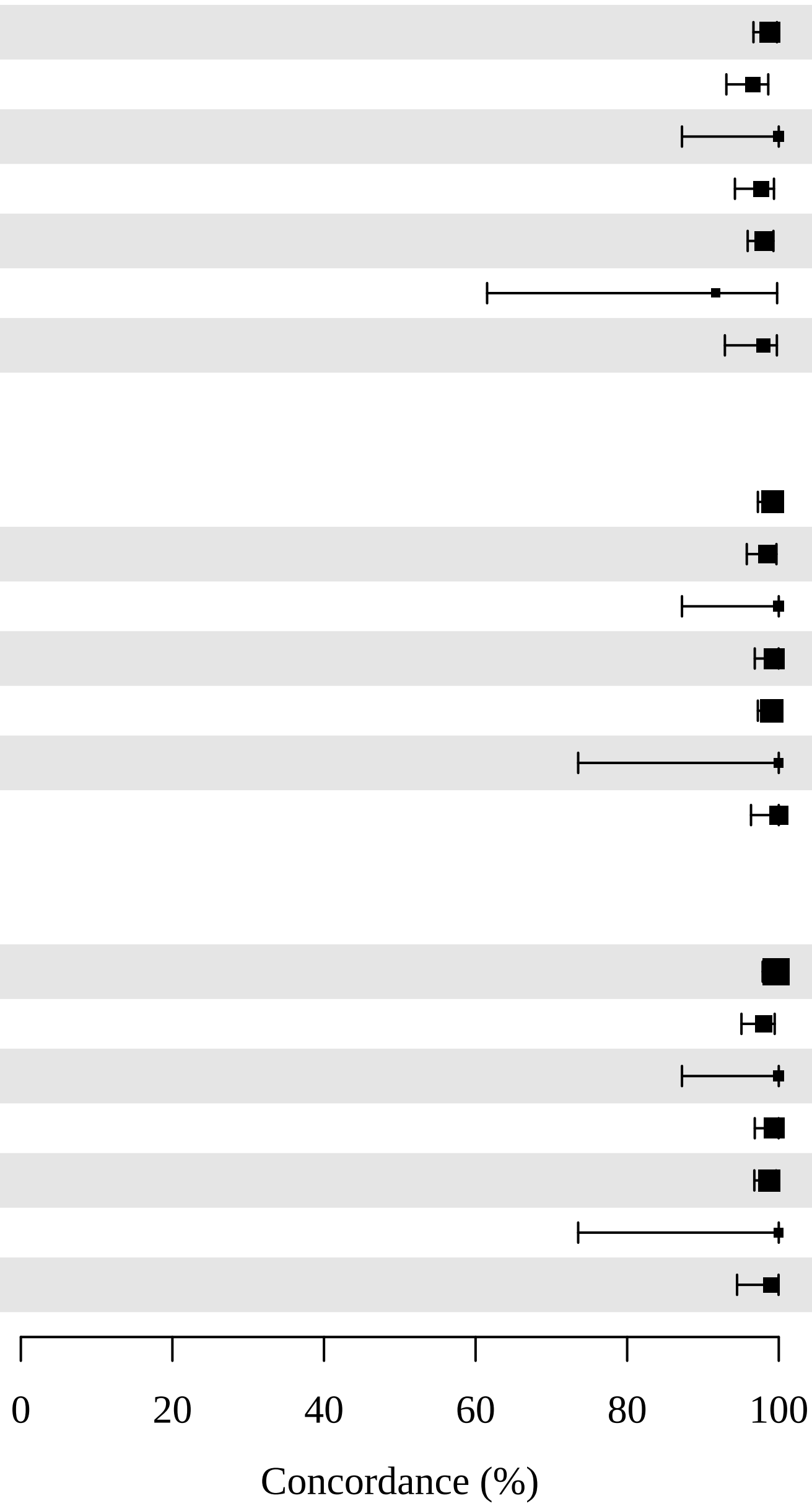

Supplement: Supplementary file 2 [file pone.0355869.s002.pdf]
