# Supplementary material for: Diagnostic agreement between Rhinoswabs and joint nose and throat swabs for the detection of influenza and SARS-CoV-2 among symptomatic ambulatory patients in Hong Kong
Source: PLoS One. 2026 Aug 11;21(8):e0355869. doi: 10.1371/journal.pone.0355869 (PMC13460600; doi:10.1371/journal.pone.0355869)
Supplement: Supplementary file 4 [file pone.0355869.s006.pdf]

Reviewer

It is a pleasure to review the manuscript entitled **"Agreement of Rhinoswabs and joint nose and throat swabs for detecting influenza and SARS-CoV-2 among symptomatic ambulatory patients in Hong Kong" (PONE-D-26-15719)**. I have carefully reviewed this manuscript. I found that it is straightforward, the sample size is reasonable (488 paired samples), the statistics are appropriate for the objective, and the conclusions are generally supported by the data. This manuscript appears scientifically sound, clinically relevant, and appropriate for publication in PLOS ONE. In particular, the manuscript is concise and well written. Nevertheless, a few minor issues should be addressed to strengthen the manuscript. After this revision, I support accepting this manuscript in PLOS ONE.

Manor's suggestion for revision

1. The study mainly reports "concordance" but does not provide sensitivity, specificity, positive predictive agreement, negative predictive agreement, and Cohen's kappa. Because NTS is effectively treated as a reference standard, reporting these metrics would strengthen diagnostic interpretation. Thus, the study would be strengthened by providing additional reporting of the sensitivity, specificity, positive/negative agreement, and Cohen's kappa statistics to provide a more comprehensive assessment of diagnostic performance.
2. The Rhinoswab was always collected AFTER rapid antigen testing and joint NTS sampling. This could reduce the amount of viral material remaining in the nose. The authors briefly mention this in the discussion. I think this deserves stronger acknowledgment. Because Rhinoswab collection was performed after other respiratory sampling procedures, the order of sampling may have influenced viral recovery. Please discuss this potential sampling-order bias in greater detail and clarify whether randomization of sampling order was considered.
3. I found a very important issue. The manuscript promotes Rhinoswab as suitable for easy or minimally invasive collection, yet all samples were collected by trained staff. Thus, the conclusions about usability and self-testing are somewhat overstated. Because all Rhinoswab specimens were collected under supervision by trained staff, the manuscript should avoid overgeneralizing the findings to unsupervised self-collection settings.
4. The authors used Ct ≤ 40 as the positivity cutoff. However, a Ct of 40 is relatively permissive. Please justify the use of a Ct of 40 as the positivity threshold and indicate whether equivocal results were observed near the cutoff.
5. The manuscript reports concordance percentages but only briefly presents confidence intervals. Please ensure that all concordance estimates include 95% confidence intervals in the main text and/or tables.
6. Please correct minor typographical and formatting errors throughout the manuscript.
