# Supplementary material for: Diagnostic agreement between Rhinoswabs and joint nose and throat swabs for the detection of influenza and SARS-CoV-2 among symptomatic ambulatory patients in Hong Kong
Source: PLoS One. 2026 Aug 11;21(8):e0355869. doi: 10.1371/journal.pone.0355869 (PMC13460600; doi:10.1371/journal.pone.0355869)
Supplement: Supplementary file 5 [file pone.0355869.s005.pdf]

PLOS One

Agreement of Rhinoswabs and joint nose and throat swabs for detecting influenza and SARS-CoV-2 among symptomatic ambulatory patients in Hong Kong

--Manuscript Draft--

**Manuscript Number:**	PONE-D-26-15719
**Article Type:**	Research Article
**Full Title:**	Agreement of Rhinoswabs and joint nose and throat swabs for detecting influenza and SARS-CoV-2 among symptomatic ambulatory patients in Hong Kong
**Short Title:**	Diagnostic agreement of Rhinoswabs and joint NTS
**Corresponding Author:**	Benjamin J. Cowling<br>The University of Hong Kong<br>Pokfulam, Hong Kong HONG KONG
**Keywords:**	Rhinoswab, nasal swab, influenza, SARS-CoV-2, diagnostic concordance
**Abstract:**	Background: Accurate detection of respiratory viruses relies on appropriate sample collection, but whether joint nose and throat sampling is necessary for high diagnostic performance or whether nose alone can provide comparable results remains uncertain.<br>Objectives: We evaluated the diagnostic agreement between joint nose and throat swabs (NTS) and Rhinoswab (RhinoMed, Cremorne, Australia), a novel anterior nasal swab for RT-PCR detection respiratory viruses among symptomatic outpatients.<br>Study design: Outpatients with acute respiratory illness were enrolled at a clinic in Hong Kong and paired joint NTS and Rhinoswab samples were tested by reverse transcription polymerase chain reaction (RT-PCR) for influenza A, influenza B, SARS-CoV-2 and human ribonuclease P (RNase P) as an internal control. Diagnostic concordance and PCR Ct values were compared between the paired samples.<br>Results: Between May 2023 and April 2024, 488 participants provided paired samples. Overall concordance between Rhinoswab and NTS was 98.0% for influenza A, 99.0% for influenza B, and 99.0% for SARS-CoV-2. Ct values from Rhinoswab were moderately correlated with NTS for all three viruses and RNase P. The majority of discordant samples had Ct values of 30 and above (16/18, 88.9%).<br>Conclusions: Rhinoswabs achieved high agreement with joint NTS for PCR detection of influenza and SARS-CoV-2. Given their minimal invasiveness Rhinoswabs are a viable alternative specimen type for respiratory virus testing.
**Order of Authors:**	Caitriona Murphy
	Samuel M. S. Cheng
	Loretta Mak
	Hau Chi So
	Dennis K M Ip
	Malik Peiris
	Benjamin J. Cowling
**Additional Information:**	
**Question**	**Response**
**Financial Disclosure**<br><br>Enter a financial disclosure statement that describes the sources of funding for the work included in this submission. Review the submission guidelines for detailed requirements. View published research articles from *PLOS ONE* for specific	Yes

examples.<br><br>This statement is required for submission and **will appear in the published article** if the submission is accepted. Please make sure it is accurate.<br><br>**Funded studies**<br>Enter a statement with the following details:<br>• Initials of the authors who received each award<br>• Grant numbers awarded to each author<br>• The full name of each funder<br>• URL of each funder website<br>• Did the sponsors or funders play any role in the study design, data collection and analysis, decision to publish, or preparation of the manuscript?<br><br>Did you receive funding for this work?	
Please add funding details.<br>as follow-up to "**Financial Disclosure**<br><br>Enter a financial disclosure statement that describes the sources of funding for the work included in this submission. Review the submission guidelines for detailed requirements. View published research articles from *PLOS ONE* for specific examples.<br><br>This statement is required for submission and **will appear in the published article** if the submission is accepted. Please make sure it is accurate.<br><br>**Funded studies**<br>Enter a statement with the following details:<br>• Initials of the authors who received each award<br>• Grant numbers awarded to each author<br>• The full name of each funder<br>• URL of each funder website<br>• Did the sponsors or funders play any role in the study design, data collection and analysis, decision to publish, or preparation of the manuscript?	This research was financially supported by a grant from the Health and Medical Research Fund, Health Bureau, the Government of the Hong Kong Special Administrative Region (grant number INF-HKU-3) and the Theme-based Research Scheme (grant number T11-712/19-N) of the Research Grants Council of the Hong Kong SAR Government.

Did you receive funding for this work?"	
Please select the country of your main research funder (please select carefully as in some cases this is used in fee calculation).<br>as follow-up to "**Financial Disclosure**<br><br>Enter a financial disclosure statement that describes the sources of funding for the work included in this submission. Review the submission guidelines for detailed requirements. View published research articles from *PLOS ONE* for specific examples.<br><br>This statement is required for submission and **will appear in the published article** if the submission is accepted. Please make sure it is accurate.<br><br>**Funded studies**<br>Enter a statement with the following details:<br>• Initials of the authors who received each award<br>• Grant numbers awarded to each author<br>• The full name of each funder<br>• URL of each funder website<br>• Did the sponsors or funders play any role in the study design, data collection and analysis, decision to publish, or preparation of the manuscript?<br><br>Did you receive funding for this work?"	HONG KONG - HK
**Competing Interests**<br><br>Use the instructions below to enter a competing interest statement for this submission. On behalf of all authors, disclose any competing interests that could be perceived to bias this work—acknowledging all financial support and any other relevant financial or non-financial competing interests.	BJC consults for AstraZeneca, Fosun Pharma, GSK, Haleon, Moderna, Novavax, Pfizer, Roche, Sanofi Pasteur, and Seqirus. The authors report no other potential conflicts of interest.

<table>
<tr><td>This statement is required for submission and will appear in the published article if the submission is accepted. Please make sure it is accurate and that any funding sources listed in your Funding Information later in the submission form are also declared in your Financial Disclosure statement.

View published research articles from PLOS ONE for specific examples.

NO authors have competing interests

Enter: The authors have declared that no competing interests exist.

Authors with competing interests

Enter competing interest details beginning with this statement:

I have read the journal's policy and the authors of this manuscript have the following competing interests: [insert competing interests here]

* typeset</td><td></td></tr>
<tr><td>Ethics Statement

Enter an ethics statement for this submission. This statement is required if the study involved:

• Human participants
• Human specimens or tissue
• Vertebrate animals or cephalopods
• Vertebrate embryos or tissues
• Field research

Write "N/A" if the submission does not require an ethics statement.

General guidance is provided below. Consult the submission guidelines for detailed instructions. Make sure that all information entered here is included in the Methods section of the manuscript.</td><td>The study protocol was approved by the Institutional Review Board of the University of Hong Kong. Written informed consent was obtained from all participants or their parents or legal guardians for minors.</td></tr>
</table>

**Format for specific study types**<br><br>**Human Subject Research (involving human participants and/or tissue)**<br>• Give the name of the institutional review board or ethics committee that approved the study<br>• Include the approval number and/or a statement indicating approval of this research<br>• Indicate the form of consent obtained (written/oral) or the reason that consent was not obtained (e.g. the data were analyzed anonymously)<br><br>**Animal Research (involving vertebrate animals, embryos or tissues)**<br>• Provide the name of the Institutional Animal Care and Use Committee (IACUC) or other relevant ethics board that reviewed the study protocol, and indicate whether they approved this research or granted a formal waiver of ethical approval<br>• Include an approval number if one was obtained<br>• If the study involved *non-human primates*, add *additional details* about animal welfare and steps taken to ameliorate suffering<br>• If anesthesia, euthanasia, or any kind of animal sacrifice is part of the study, include briefly which substances and/or methods were applied<br><br>**Field Research**<br><br>Include the following details if this study involves the collection of plant, animal, or other materials from a natural setting:<br>• Field permit number<br>• Name of the institution or relevant body that granted permission	
**Data Availability**<br><br>Authors are required to make all data underlying the findings described fully available, without restriction, and from the time of publication. PLOS allows rare exceptions to address legal and ethical concerns. See the PLOS Data Policy and FAQ for detailed information.	Yes - all data are fully available without restriction

A Data Availability Statement describing where the data can be found is required at submission. Your answers to this question constitute the Data Availability Statement and **will be published in the article**, if accepted.<br><br>**Important:** Stating 'data available on request from the author' is not sufficient. If your data are only available upon request, select 'No' for the first question and explain your exceptional situation in the text box.<br><br>Do the authors confirm that all data underlying the findings described in their manuscript are fully available without restriction?	
**Describe where the data may be found in full sentences. If you are copying our sample text, replace any instances of XXX with the appropriate details.**<br><br>• If the data are **held or will be held in a public repository**, include URLs, accession numbers or DOIs. If this information will only be available after acceptance, indicate this by ticking the box below. For example: *All XXX files are available from the XXX database (accession number(s) XXX, XXX.).*<br>• If the data are all contained **within the manuscript and/or Supporting Information files**, enter the following: *All relevant data are within the manuscript and its Supporting Information files.*<br>• If neither of these applies but you are able to provide **details of access elsewhere**, with or without limitations, please do so. For example:<br><br>*Data cannot be shared publicly because of [XXX]. Data are available from the XXX Institutional Data Access / Ethics Committee (contact via XXX) for researchers who meet the criteria for access to confidential data.*<br><br>*The data underlying the results presented in the study are available*	It will be made available upon acceptance in GitHub.

from (include the name of the third party and contact information or URL).<br>• This text is appropriate if the data are owned by a third party and authors do not have permission to share the data.<br><br>* typeset	
Additional data availability information:	

Manuscript

Click here to access/download;Manuscript;rhino_concordance.docx

Agreement of Rhinoswabs and joint nose and throat swabs for detecting influenza and SARS-CoV-2 among symptomatic ambulatory patients in Hong Kong.

Caitriona Murphy[1], Samuel M. S. Cheng[1], Loretta Mak[1], Hau Chi So[1], Dennis K. M. Ip[1], Malik Peiris[1,2], Benjamin J. Cowling[1]

Affiliations:

1. World Health Organization Collaborating Centre for Infectious Disease Epidemiology and Control, School of Public Health, The University of Hong Kong, Hong Kong Special Administrative Region, China

2. Centre of Immunology and Infection, Hong Kong Science and Technology Park, Hong Kong Special Administrative Region, China

Correspondence: bcowling@hku.hk

Running head: Diagnostic agreement of Rhinoswabs and joint NTS

Word count (abstract): 220 (max 250)

Word count (main text): 1248 (max 1250)

ABSTRACT

Background: Accurate detection of respiratory viruses relies on appropriate sample collection, but whether joint nose and throat sampling is necessary for high diagnostic performance or whether nose alone can provide comparable results remains uncertain.

Objectives: We evaluated the diagnostic agreement between joint nose and throat swabs (NTS) and Rhinoswab (RhinoMed, Cremorne, Australia), a novel anterior nasal swab for RT-PCR detection respiratory viruses among symptomatic outpatients.

Study design: Outpatients with acute respiratory illness were enrolled at a clinic in Hong Kong and paired joint NTS and Rhinoswab samples were tested by reverse transcription polymerase chain reaction (RT-PCR) for influenza A, influenza B, SARS-CoV-2 and human ribonuclease P (RNase P) as an internal control. Diagnostic concordance and PCR Ct values were compared between the paired samples.

Results: Between May 2023 and April 2024, 488 participants provided paired samples. Overall concordance between Rhinoswab and NTS was 98.0% for influenza A, 99.0% for influenza B, and 99.0% for SARS-CoV-2. Ct values from Rhinoswab were moderately correlated with NTS for all three viruses and RNase P. The majority of discordant samples had Ct values of 30 and above (16/18, 88.9%).

Conclusions: Rhinoswabs achieved high agreement with joint NTS for PCR detection of influenza and SARS-CoV-2. Given their minimal invasiveness Rhinoswabs are a viable alternative specimen type for respiratory virus testing.

Keywords: Rhinoswab, nasal swab, influenza, SARS-CoV-2, diagnostic concordance

BACKGROUND

Accurate and timely detection of influenza and SARS-CoV-2 is important for surveillance and clinical management. Molecular testing by polymerase chain reaction (PCR) is the gold standard but its accuracy is contingent on the quality of the specimen collected which is influenced by the anatomical site of active viral replication sampled. In Hong Kong, diagnostic testing is typically carried out using nasopharyngeal swabs or joint nose and throat swabs (NTS). In contrast to nasopharyngeal swabbing, which requires deep insertion into the nasal cavity by a trained professional, joint NTS is less invasive and suitable for self-collection. However, an evaluation of the UK COVID-19 National Testing Programme observed a preference for nose-only swabbing compared to joint NTS, reporting it was easier to perform and less uncomfortable [1]. The Rhinoswab™ was developed by RhinoMed (Cremorne, Australia) in 2020 [2,3] aiming to be minimally invasive by collecting nasal discharge from the anterior nares.

OBJECTIVES

We aimed to estimate the concordance between Rhinoswabs and joint NTS for detecting influenza and SARS-CoV-2 via RT-PCR among symptomatic outpatients in Hong Kong.

STUDY DESIGN

Study participants

We utilised data from an ongoing outpatient surveillance study in Hong Kong, between 03/05/2023 and 29/04/2024. Eligible patients were aged ≥6 months and were seeking medical care for a febrile acute respiratory illness, defined as the presence of ≥2 respiratory symptoms within 3 days of symptom onset. Written informed consent was obtained from all participants or

their legal guardians. A questionnaire was administered to collect vaccination history and demographics. The study protocol was approved by the Institutional Review Board of the University of Hong Kong. Written informed consent was obtained from all participants or their parents or legal guardians for minors.

Specimen collection and laboratory testing

All participants were first sampled using rapid antigen tests (unrelated to this study), followed by a joint NTS (CLASSIQSwabs™, Copan) and then by the Rhinoswab as participants could opt out of being sampled using a Rhinoswab. A demonstration video was shown and a junior version of the Rhinoswab was available for children (Figure S1). For participants that agreed to provide a Rhinoswab sample, collection was performed by study staff according to manufacturers' instructions. Swabs were inserted into the anterior nares and left for 15 seconds while the participant breathed normally and then moved back and forth in the nostril for an additional 15 seconds. After removal, each loop of the swab was snapped off into the collection tube at predefined break points. All swabs were transported to the laboratory in viral transport media and tested for influenza A, influenza B, and SARS-CoV-2 using a ViiA7 RT-PCR system (ThermoFisher). RNA extraction and RT-PCR targets and conditions have been described previously [4]. A cycle threshold (Ct) value of <40 was considered positive.

Statistical analysis

Participants were included if they had paired joint NTS and Rhinoswab samples. Agreement was assessed by calculating concordance, defined as the percentage of paired swabs with identical results (positive or negative). Concordance was evaluated by age, time since the onset of

symptoms and vaccination status (received influenza or COVID-19 vaccination within a year before seeking medical care). In addition, the correlation between the Ct values for both swabs was assessed using Pearson's correlation coefficients. Analyses were performed using R version 4.0.2 (R Foundation for Statistical Computing, Vienna, Austria).

RESULTS

During the study period, 531 participants were enrolled and invited to provide paired samples, of whom 488 (91.9%) provided paired samples, while 43 (8.1%) opted out. Of those that opted out, 16/43 (37.2%) were unwilling after viewing the demonstration video. Of the 488 Rhinoswabs collected, 246 (50.4%) were Rhinoswab junior. The mean age was 25 years and the majority of participants were female (55.5%) (Table 1).

Overall agreement between Rhinoswab and joint NTS across all samples was 96.3%. Influenza A was detected in 136 participants by both joint NTS and Rhinoswab, with a concordance of 98.0% (95% CI: 96.3% to 99.0%) and 2.0% with discordant results. There were 42 influenza B and 32 SARS-CoV-2 positives by both swabs respectively (Table 1). The concordance for detecting influenza B and SARS-CoV-2 were the same at 99.0% (95% CI: 97.6% to 99.7%). Concordance was similar among different age groups, time since onset and vaccination status (Figure S2).

The Ct values from Rhinoswabs were significantly positively correlated with those from joint NTS for all three viruses and the internal control, human RNase P (Figure 1 panels A-D). Correlations coefficients were 0.76 for influenza A, 0.818 for influenza B, and 0.729 for SARS-

CoV-2, while human RNase P showed a moderate correlation (r = 0.616). When the difference between Ct values (Rhinoswab minus NTS) was plotted against the NTS Ct value there was no evidence of substantially different Rhinoswab performance for higher or lower Ct values (Figure 1 panels E-H). The majority of discordant samples (16/18, 88.9%) had Ct values of ≥30 and occurred within 24 hours of symptom onset (Table 1).

DISCUSSION

Rhinoswabs had a high concordance with joint NTS for detecting influenza A (98.0%), influenza B (99.0%), and SARS-CoV-2 (99.0%). This is higher than the 95.2% concordance reported for SARS-CoV-2 estimated among symptomatic patients in Australia [2]. Two other studies have evaluated Rhinoswabs, observing a positive percent agreement of 96.2% for detecting six viruses in a hospital setting [3] and sensitivity of 95% in patients with high viral loads (Ct < 20) compared to combined oro-nasopharyngeal swabs in an emergency department population [5]. However, these metrics are not directly comparable to the concordance reported here. Discordance was among samples with Ct values of ≥30, similar to previous literature for non-nasopharyngeal samples at low viral loads [7]. This may not affect case detection when transmissibility is greatest (higher viral loads) [8].

We observed a lower (compared to influenza and SARS-CoV-2) correlation for RNase P between Rhinoswab and joint NTS Ct values, which could reflect variability in the amount of cellular material collected by the two swab types. However, differences in host-cell yield do not necessarily translate into poorer viral RNA detection. For example, a comparison of oropharyngeal, nasopharyngeal and combined swabs observed varied RNase P and total human

RNA levels but these differences did not correlate with viral load measured by PCR [9]. Similar patterns were reported for nasal and saliva specimens, suggesting more variable host-cell recovery while still maintaining comparable SARS-CoV-2 detection to nasopharyngeal swabs [10].

Rhinoswabs in this study were the third sequential sampling of the anterior nares. Despite this, performance remained comparable to joint NTS, suggesting robustness to depletion of viral material that may have occurred in the nose. This finding is similar to studies that carried out sequential sampling to compare self-collected samples followed by a healthcare worker collecting swabs, finding that self-collected nasal and mid-turbinate swabs yielded viral loads equivalent to or even higher than healthcare-worker-collected nasopharyngeal swabs [11,12].

Limitations include sample collection early in the course of infection, when viral loads are typically highest and therefore, the high concordance observed may not generalise to patients with lower viral loads. Rhinoswab collection was supervised by trained staff, and we did not evaluate self-collection. Finally, the study was conducted among symptomatic outpatients in Hong Kong, and its applicability to inpatient settings or to asymptomatic screening in the community requires further evaluation.

To conclude, Rhinoswabs demonstrated a high concordance with joint NTS for the detection of influenza and SARS-CoV-2 in symptomatic outpatients. Given Rhinoswabs only sample the nose and are child friendly, they can be a practical alternative for respiratory virus testing in outpatient settings.

ACKNOWLEDGMENTS

The authors thank colleagues at St Teresa's Hospital for facilitating participant enrolment. The authors thank Julie Au for technical support.

FUNDING

This research was financially supported by a grant from the Health and Medical Research Fund, Health Bureau, the Government of the Hong Kong Special Administrative Region (grant number INF-HKU-3) and the Theme-based Research Scheme (grant number T11-712/19-N) of the Research Grants Council of the Hong Kong SAR Government.

POTENTIAL CONFLICTS OF INTEREST

BJC consults for AstraZeneca, Fosun Pharma, GSK, Haleon, Moderna, Novavax, Pfizer, Roche, Sanofi Pasteur, and Seqirus. The authors report no other potential conflicts of interest.

REFERENCES

[1] M.E. Futschik, R. Kulasegaran-Shylini, E. Blandford, S. Harper, D. Chapman, E. Turek, S. Agrawal, V. Phillips, H. Fordham, L. Chan, M. Kidd, A. Dodgson, P.E. Klapper, M. Sudhanva, R. Vipond, S. Hopkins, T. Peto, S. Tunkel, T. Fowler, Effectiveness and user experience of nose and throat swabbing techniques for SARS-CoV-2 detection: results from the UK COVID-19 National Testing Programme, BMC Glob Public Health 3 (2025) 5.

[2] A. Crowe, Y.P. Lorenzo, D. Jardine, K. Visvanathan, Evaluation of a novel anterior nasal swab for the detection of SARS-CoV-2, J Virol Methods 316 (2023) 114713.

[3] S. Tosif, L.-Y. Lee, J. Nguyen, I. Overmars, C. Selman, A.C. Grobler, A. McMinn, G. Waller, S. McNab, T. Jarvis, A. Steer, F.E. Babl, A. Daley, N.W. Crawford, A novel anterior nasal swab to detect respiratory viruses: a prospective study of diagnostic accuracy, BMC Pediatr 23 (2023) 201.

[4] C. Murphy, L. Mak, S.M.S. Cheng, G.Y.Z. Liu, A.M.C. Chun, K.K.Y. Leung, N.Y.W. Sum, E. Poukka, M. Peiris, B.J. Cowling, Diagnostic performance of multiplex lateral flow tests in ambulatory patients with acute respiratory illness, Diagn Microbiol Infect Dis 110 (2024) 116421.

[5] J.G.M. Koeleman, S. Mol, H. Brand, D.S.Y. Ong, Evaluation of a New Standardized Nasal Sampling Method for Detection of SARS-CoV-2 RNA via RT-PCR, Microorganisms 12 (2024). https://doi.org/10.3390/microorganisms12010210.

[6] C.A. Cismaru, S. Chira, G.L. Cismaru, A.M. Nutu, M.G. Netea, I. Berindan-Neagoe, Assessment of the frequency of coughing and sneezing triggered by nasopharyngeal swabbing in the pandemic setting, Sci Rep 12 (2022) 10874.

[7] R.A. Lee, J.C. Herigon, A. Benedetti, N.R. Pollock, C.M. Denkinger, Performance of Saliva, Oropharyngeal Swabs, and Nasal Swabs for SARS-CoV-2 Molecular Detection: a Systematic Review and Meta-analysis, J Clin Microbiol 59 (2021). https://doi.org/10.1128/JCM.02881-20.

[8] Y. Tsujimoto, J. Terada, M. Kimura, A. Moriya, A. Motohashi, S. Izumi, K. Kawajiri, K. Hakkaku, M. Morishita, S. Saito, H. Takumida, H. Watanabe, A. Tsukada, C. Morita, Y. Yamaguchi, T. Katsuno, Y. Kusaba, K. Sakamoto, M. Hashimoto, M. Suzuki, J. Takasaki, M. Hojo, T. Miyoshi-Akiyama, H. Sugiyama, Diagnostic accuracy of nasopharyngeal swab, nasal swab and saliva swab samples for the detection of SARS-CoV-2 using RT-PCR, Infect Dis (Lond) 53 (2021) 581–589.

[9] F. Ibrahim, A. Natasha, A. Yasmon, C.T. Rizal, Fithriyah, A. Karuniawati, Y.R. Saharman, P. Sudarmono, Evaluation of SARS-CoV-2 quantification from oropharyngeal swabs, nasopharyngeal swabs, and naso-oropharyngeal swabs: A cross-sectional study, Heliyon 10 (2024) e28647.

[10] S.A. Kiryanov, T.A. Levina, V.V. Kadochnikova, M.V. Konopleva, A.P. Suslov, D.Y. Trofimov, Clinical Evaluation of Nasopharyngeal, Oropharyngeal, Nasal Swabs, and Saliva for the Detection of SARS-CoV-2 by Direct RT-PCR, Diagnostics (Basel) 12 (2022). https://doi.org/10.3390/diagnostics12051091.

[11] Y.-P. Tu, R. Jennings, B. Hart, G.A. Cangelosi, R.C. Wood, K. Wehber, P. Verma, D. Vojta, E.M. Berke, Swabs Collected by Patients or Health Care Workers for SARS-CoV-2 Testing, N Engl J Med 383 (2020) 494–496.

[12] M.C. Wehrhahn, J. Robson, S. Brown, E. Bursle, S. Byrne, D. New, S. Chong, J.P. Newcombe, T. Siversten, N. Hadlow, Self-collection: An appropriate alternative during the

SARS-CoV-2 pandemic, J Clin Virol 128 (2020) 104417.

Table 1: Study participant characteristics by virus detected by both Rhinoswab and Nasal and throat swab.

**Characteristic**	**Influenza A (n = 136)**	**Influenza B (n = 42)**	**SARS-CoV-2 (n = 32)**	**Negative (n = 260)**	**Discordant (n = 18)**	**Overall (n = 488)**
Age, Mean (SD)	25.6 (20.3)	25.1 (15.9)	41.7 (22.1)	22.4 (20.3)	29.2 (18.5)	25.1 (20.5)
Male	62 (45.6%)	23 (54.8%)	10 (31.3%)	117 (45.0%)	5 (27.8%)	217 (44.5%)
Chronic condition	12 (8.8%)	2 (4.8%)	5 (15.6%)	28 (10.8%)	3 (16.7%)	50 (10.2%)
**Vaccinated within a year**						
Influenza vaccine	41 (30.1%)	5 (11.9%)	9 (28.1%)	111 (42.7%)	5 (27.8%)	171 (35.0%)
COVID-19 vaccine	6 (4.4%)	0 (0%)	1 (3.1%)	4 (1.5%)	1 (5.6%)	12 (2.5%)
**Symptom onset**						
≤ 24 hours	71 (52.2%)	12 (28.6%)	26 (81.3%)	132 (50.8%)	12 (66.7%)	253 (51.8%)
> 24 to 47 hours	27 (19.9%)	8 (19.0%)	3 (9.4%)	38 (14.6%)	3 (16.7%)	79 (16.2%)
> 48 to 72 hours	38 (27.9%)	22 (52.4%)	3 (9.4%)	90 (34.6%)	3 (16.7%)	156 (32.0%)

FIGURE LEGENDS

Figure 1: Correlation of cycle threshold (Ct) values between joint NT swabs and Rhinoswabs for influenza A, influenza B, SARS-CoV-2 and the internal control, human ribonuclease P (top row). The bottom row shows the difference between the swabs Ct-values against the NTS Ct-value (y-axis) versus the NTS Ct value (x-axis).

Figure 1

Click here to access/download;Figure;Figure 1.pdf

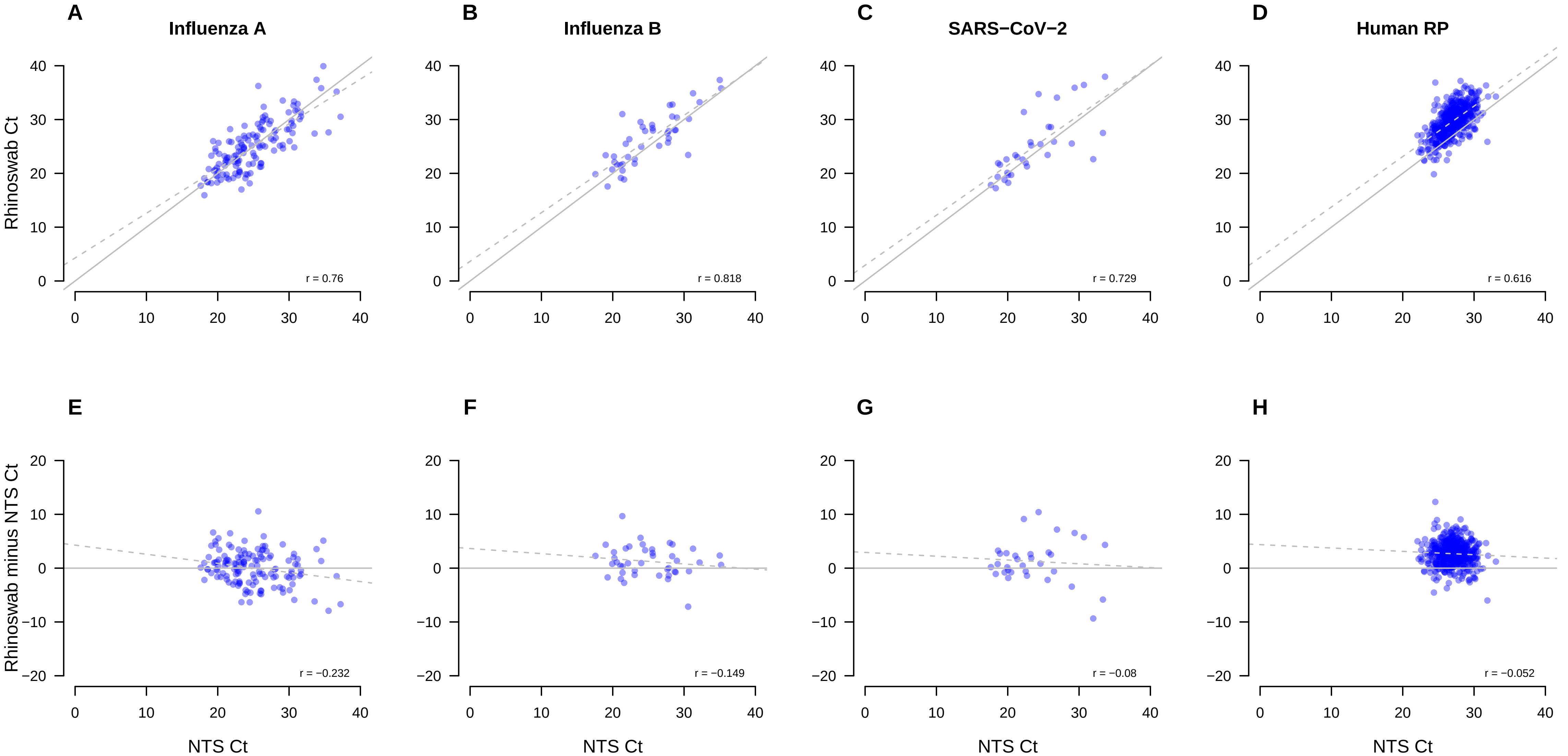

Click here to access/download
Supporting Information
Supplementary information.pdf
