# Supplementary material for: Diagnostic agreement between Rhinoswabs and joint nose and throat swabs for the detection of influenza and SARS-CoV-2 among symptomatic ambulatory patients in Hong Kong
Source: PLoS One. 2026 Aug 11;21(8):e0355869. doi: 10.1371/journal.pone.0355869 (PMC13460600; doi:10.1371/journal.pone.0355869)
Supplement: Supplementary file 5 [file pone.0355869.s003.docx]

S1 Table. Number of concordant and discordant results between Rhinoswab and joint NTS, with mean cycle threshold values for each category.

| **Influenza A** | | | **Influenza B** | | | **SARS-CoV-2** | | |
| --- | --- | --- | --- | --- | --- | --- | --- | --- |
| No. samples | Rhinoswab | Joint NTS | No. samples | Rhinoswab | Joint NTS | No. samples | Rhinoswab | Joint NTS |
| 136 | 25.0 | 25.0 | 42 | 26.3 | 25.1 | 32 | 25.0 | 23.7 |
| 6 | - | 31.0 | 4 | - | 33.3 | 3 | - | 34.3 |
| 4 | 37.6 | - | 1 | 39.5 | - | 2 | 31.8 | - |
| 342 | - | - | 441 | - | - | 451 | - | - |
