# Supplementary material for: Diagnostic agreement between Rhinoswabs and joint nose and throat swabs for the detection of influenza and SARS-CoV-2 among symptomatic ambulatory patients in Hong Kong
Source: PLoS One. 2026 Aug 11;21(8):e0355869. doi: 10.1371/journal.pone.0355869 (PMC13460600; doi:10.1371/journal.pone.0355869)
Supplement: Supplementary file 6 [file pone.0355869.s004.docx]

S2 Table. Diagnostic performance of Rhinoswab compared to joint NTS by virus.

| **Virus** | **Sensitivity**  **(%, 95%CI)** | **Specificity**  **(%, 95%CI)** | **PPV**  **(%, 95%CI)** | **NPV**  **(%, 95%CI)** |
| --- | --- | --- | --- | --- |
| Influenza A | 95.8 (91.0-98.4) | 98.8 (97.1-99.7) | 97.1 (92.8-99.2) | 98.3 (96.3-99.4) |
| Influenza B | 91.3 (79.2-97.6) | 99.8 (98.7-100) | 97.7 (87.7-99.9) | 99.1 (97.7-99.8) |
| SARS-CoV-2 | 91.4 (76.9-98.2) | 99.6 (98.4-99.9) | 94.1 (80.3-99.3) | 99.3 (98.1-99.9) |
