# Supplementary material for: Diagnostic agreement between Rhinoswabs and joint nose and throat swabs for the detection of influenza and SARS-CoV-2 among symptomatic ambulatory patients in Hong Kong
Source: PLoS One. 2026 Aug 11;21(8):e0355869. doi: 10.1371/journal.pone.0355869 (PMC13460600; doi:10.1371/journal.pone.0355869)
Supplement: Supplementary file 8 [file pone.0355869.s008.docx]

**Journal Requirements:**

1. When submitting your revision, we need you to address these additional requirements.

Please ensure that your manuscript meets PLOS ONE's style requirements, including those for file naming. The PLOS ONE style templates can be found at

<https://journals.plos.org/plosone/s/file?id=wjVg/PLOSOne_formatting_sample_main_body.pdf> and

<https://journals.plos.org/plosone/s/file?id=ba62/PLOSOne_formatting_sample_title_authors_affiliations.pdf>

Thank you. The manuscript has been edited accordingly.

2. Thank you for stating in your Funding Statement:

This research was financially supported by a grant from the Health and Medical Research Fund, Health Bureau, the Government of the Hong Kong Special Administrative Region (grant number INF-HKU-3) and the Theme-based Research Scheme (grant number T11-712/19-N) of the Research Grants Council of the Hong Kong SAR Government.

Please provide an amended statement that declares *all* the funding or sources of support (whether external or internal to your organization) received during this study, as detailed online in our guide for authors at <http://journals.plos.org/plosone/s/submit-now>.  Please also include the statement “There was no additional external funding received for this study.” in your updated Funding Statement.

Please include your amended Funding Statement within your cover letter. We will change the online submission form on your behalf.

We have updated the statement to include that there was no additional funding received for this study.

This research was financially supported by a grant received by BJC from the Health and Medical Research Fund, Health Bureau, the Government of the Hong Kong Special Administrative Region (grant number INF-HKU-3) and the Theme-based Research Scheme (grant number T11-712/19-N) of the Research Grants Council of the Hong Kong SAR Government. There was no additional external funding received for this study. The funders had no role in study design, data collection and analysis, decision to publish, or preparation of the manuscript.

3. Thank you for stating the following financial disclosure:

This research was financially supported by a grant from the Health and Medical Research Fund, Health Bureau, the Government of the Hong Kong Special Administrative Region (grant number INF-HKU-3) and the Theme-based Research Scheme (grant number T11-712/19-N) of the Research Grants Council of the Hong Kong SAR Government.

Please state what role the funders took in the study.  If the funders had no role, please state: "The funders had no role in study design, data collection and analysis, decision to publish, or preparation of the manuscript."

If this statement is not correct you must amend it as needed.

Please include this amended Role of Funder statement in your cover letter; we will change the online submission form on your behalf.

The funders had no role and therefore the above statement has been included in the manuscript.

4. Thank you for stating the following in the Competing Interests section:

BJC consults for AstraZeneca, Fosun Pharma, GSK, Haleon, Moderna, Novavax, Pfizer, Roche, Sanofi Pasteur, and Seqirus. The authors report no other potential conflicts of interest.

We note that one or more of the authors are employed by a commercial company.

a. Please provide an amended Funding Statement declaring this commercial affiliation, as well as a statement regarding the Role of Funders in your study. If the funding organization did not play a role in the study design, data collection and analysis, decision to publish, or preparation of the manuscript and only provided financial support in the form of authors' salaries and/or research materials, please review your statements relating to the author contributions, and ensure you have specifically and accurately indicated the role(s) that these authors had in your study. You can update author roles in the Author Contributions section of the online submission form.

Please also include the following statement within your amended Funding Statement.

“The funder provided support in the form of salaries for authors [insert relevant initials], but did not have any additional role in the study design, data collection and analysis, decision to publish, or preparation of the manuscript. The specific roles of these authors are articulated in the ‘author contributions’ section.”

If your commercial affiliation did play a role in your study, please state and explain this role within your updated Funding Statement.

The Centre for Immunology and Infection provided support in the form of salaries for authors (MP) but did not have any additional role in the study design, data collection and analysis, decision to publish, or preparation of the manuscript. The specific roles of these authors are articulated in the ‘author contributions’ section.

b. Please also provide an updated Competing Interests Statement declaring this commercial affiliation along with any other relevant declarations relating to employment, consultancy, patents, products in development, or marketed products, etc.

Within your Competing Interests Statement, please confirm that this commercial affiliation does not alter your adherence to all PLOS ONE policies on sharing data and materials by including the following statement: "This does not alter our adherence to  PLOS ONE policies on sharing data and materials.” (as detailed online in our guide for authors <http://journals.plos.org/plosone/s/competing-interests>) . If this adherence statement is not accurate and  there are restrictions on sharing of data and/or materials, please state these. Please note that we cannot proceed with consideration of your article until this information has been declared.

Please include both an updated Funding Statement and Competing Interests Statement in your cover letter. We will change the online submission form on your behalf.

There is no relevant commercial interest for C2I. This does not alter our adherence to PLOS ONE policies on sharing data and materials.

5. Thank you for stating the following in the Competing Interests section:

BJC consults for AstraZeneca, Fosun Pharma, GSK, Haleon, Moderna, Novavax, Pfizer, Roche, Sanofi Pasteur, and Seqirus. The authors report no other potential conflicts of interest.

Please confirm that this does not alter your adherence to all PLOS ONE policies on sharing data and materials, by including the following statement: "This does not alter our adherence to  PLOS ONE policies on sharing data and materials.” (as detailed online in our guide for authors <http://journals.plos.org/plosone/s/competing-interests>).  If there are restrictions on sharing of data and/or materials, please state these. Please note that we cannot proceed with consideration of your article until this information has been declared.

Please include your updated Competing Interests statement in your cover letter; we will change the online submission form on your behalf.

Thank you. We have added the statement: “This does not alter our adherence to PLOS ONE policies on sharing data and materials”. The data has also now been made available on GitHub.

6. In the online submission form, you indicated that your data will be submitted to a repository upon acceptance.  We strongly recommend all authors deposit their data before acceptance, as the process can be lengthy and hold up publication timelines. Please note that, though access restrictions are acceptable now, your entire minimal  dataset will need to be made freely accessible if your manuscript is accepted for publication. This policy applies to all data except where public deposition would breach compliance with the protocol approved by your research ethics board. If you are unable to adhere to our open data policy, please kindly revise your statement to explain your reasoning and we will seek the editor's input on an exemption.

Thank you. The data have also now been made available on GitHub.

7. We note that Figure S1 A in your submission contain copyrighted images. All PLOS content is published under the Creative Commons Attribution License (CC BY 4.0), which means that the manuscript, images, and Supporting Information files will be freely available online, and any third party is permitted to access, download, copy, distribute, and use these materials in any way, even commercially, with proper attribution. For more information, see our copyright guidelines: <http://journals.plos.org/plosone/s/licenses-and-copyright>.

We require you to either present written permission from the copyright holder to publish these figures specifically under the CC BY 4.0 license, or remove the figures from your submission:

a. You may seek permission from the original copyright holder of Figure S1 A to publish the content specifically under the CC BY 4.0 license.

We recommend that you contact the original copyright holder with the Content Permission Form (<http://journals.plos.org/plosone/s/file?id=7c09/content-permission-form.pdf>) and the following text:

“I request permission for the open-access journal PLOS ONE to publish XXX under the Creative Commons Attribution License (CCAL) CC BY 4.0 (<http://creativecommons.org/licenses/by/4.0/>). Please be aware that this license allows unrestricted use and distribution, even commercially, by third parties. Please reply and provide explicit written permission to publish XXX under a CC BY license and complete the attached form.”

Please upload the completed Content Permission Form or other proof of granted permissions as an "Other" file with your submission.

In the figure caption of the copyrighted figure, please include the following text: “Reprinted from [ref] under a CC BY license, with permission from [name of publisher], original copyright [original copyright year].”

For Figure S1 A, we used our own stock of swabs and took the picture ourselves and this picture is not copyrighted.

b. If you are unable to obtain permission from the original copyright holder to publish these figures under the CC BY 4.0 license or if the copyright holder’s requirements are incompatible with the CC BY 4.0 license, please either i) remove the figure or ii) supply a replacement figure that complies with the CC BY 4.0 license. Please check copyright information on all replacement figures and update the figure caption with source information. If applicable, please specify in the figure caption text when a figure is similar but not identical to the original image and is therefore for illustrative purposes only.

8. We note that Figure S1 B includes an image of a patient in the study.

As per the PLOS ONE policy (<http://journals.plos.org/plosone/s/submission-guidelines#loc-human-subjects-research>) on papers that include identifying, or potentially identifying, information, the individual(s) or parent(s)/guardian(s) must be informed of the terms of the PLOS open-access (CC-BY) license and provide specific permission for publication of these details under the terms of this license. Please download the Consent Form for Publication in a PLOS Journal (<http://journals.plos.org/plosone/s/file?id=8ce6/plos-consent-form-english.pdf>). The signed consent form should not be submitted with the manuscript, but should be securely filed in the individual's case notes. Please amend the methods section and ethics statement of the manuscript to explicitly state that the patient/participant has provided consent for publication: “The individual in this manuscript has given written informed consent (as outlined in PLOS consent form) to publish these case details”.

If you are unable to obtain consent from the subject of the photograph, you will need to remove the figure and any other textual identifying information or case descriptions for this individual.

Thank you. The picture is of the manuscripts first author, not a study participant. We have consent and as such have updated the ethics statement of the manuscript.

The ethics statement now reads:

“The study protocol was approved by the Institutional Review Board of the University of Hong Kong. Written informed consent was obtained from all participants or their legal guardians for minors. The individual pictured in this manuscript has given written informed consent to publish the image.”

9. Please include captions for your Supporting Information files at the end of your manuscript, and update any in-text citations to match accordingly. Please see our Supporting Information guidelines for more information: <http://journals.plos.org/plosone/s/supporting-information>.

The Supporting Information section has now been added before the acknowledgements section of the paper, listing the captions of the supplementary figures and tables as well as linking the GitHub dataset and script.

<https://github.com/caitmurphy/rhinoswab_concordance>

10. If the reviewer comments include a recommendation to cite specific previously published works, please review and evaluate these publications to determine whether they are relevant and should be cited. There is no requirement to cite these works unless the editor has indicated otherwise.

Thank you. The reviewers did not indicate previously published works to reference.

11. Please review your reference list to ensure that it is complete and correct. If you have cited papers that have been retracted, please include the rationale for doing so in the manuscript text, or remove these references and replace them with relevant current references. Any changes to the reference list should be mentioned in the rebuttal letter that accompanies your revised manuscript. If you need to cite a retracted article, indicate the article’s retracted status in the References list and also include a citation and full reference for the retraction notice.

Thank you. The references have been edited to ensure all of them contain their doi as requested. We have not referenced any retracted papers or preprints.

**Reviewer #1:**

The manuscript addresses an important and clinically relevant topic regarding minimally invasive respiratory virus sampling methods.
The paired-sample design and use of RT-PCR strengthen the technical quality of the study.
The results demonstrate high concordance between Rhinoswabs and joint nose and throat swabs, supporting the potential utility of Rhinoswabs in outpatient settings.
The manuscript is generally well written and logically organized.

Thank you.

**1.1** Statistical analysis is appropriate; however, inclusion of Cohen’s kappa statistics, sensitivity, specificity, and predictive values would provide a more comprehensive assessment of diagnostic agreement.

Thank you. We have added Cohen’s kappa statistics to the main text and a supplementary table containing the other diagnostic performance metrics.

**1.2** Additional discussion regarding applicability in asymptomatic individuals and unsupervised self-collection settings would strengthen the manuscript.

Thank you. We have now mentioned that the concordance observed in our study may not generalize to those with lower viral loads or asymptomatic individuals and included further discussion on collection methods in the limitations paragraph of the discussion.

**Reviewer #2:** The manuscript by Murphy et al addresses a practical question for clinical diagnostics. The authors provide a clear evaluation of a less invasive Rhinoswab swabbing method compared with standard practices. The manuscript is methodologically sound. The results are useful in clinical practice. I have several comments to improve the clarity and robustness before acceptance.
Thank you.

There were 18 discordant samples stated in Table 1. Please specify which swab yielded the positive result in these cases. It will be useful to know whether the Rhinoswab failed to detect viruses found by the NTS, or if the NTS missed viruses detected by the Rhinoswab. This also helps to address the next comment.

Negative cases are the majority here, which could skew the concordant value. For example, there are 32 positive SARS-CoV-2 cases, which means 456 negative cases. Let’s assume that Rhioswab couldn’t get any SARS-CoV-2 virus at all, thus it would only generate negative cases. In this scenario, the concordant value could still be as high as 456/488 = 93.4%. Please comment on this in the Discussion session.

Thank you. We have added this point to the first paragraph of the discussion (lines 155 to 162).

**2.1.** Please consider using RT-qPCR since Ct values are obtained from qPCR.

Yes, thank you. We have changed that throughout the text.

**2.2**. “Written informed consent..” is stated twice at lines 64 and 67.

Thank you. We removed the first statement.

**2.3.** How is the Ct value determined? Baseline-Threshold or regression method?

The Ct value is determined using Baseline-Threshold.

**2.4.** Have any abnormal amplification curves been observed? If yes, how were the abnormal curves handled? This information should be included in the manuscript.

Thank you. No abnormal curves were observed.

**2.5.** It will also be useful to detail the discordant cases (see above). Such as, how many have tested positive by Rhinoswab, but negative by joint NTS?

Thank you. We have since added a supplementary table showing the distribution of the concordant and discordant samples by swab types and referred to it in the results section.

**2.6.** Figure 1 legend: Please use NTS for consistency. Please indicate that the r is Pearson’s correlation (I assume).

Thank you. Edited the figure legend accordingly.

**2.7**. Figure 1: There are two linear regression lines, a solid and a dashed one, in the figure. What are they?

Thank you, we have edited the figure legend accordingly. The solid lines mark the perfect agreement while the dotted lines are the fitted regression lines.

**2.8.** Figure 1: I assume in the figure only positive cases are plotted. Please make this clear in the legend.

Thank you. Edited the figure legend accordingly.

**2.9**. Figure 1: Please consider indicating the discordant samples, maybe with a different color, to support the conclusion at line 114.

Thank you. Only samples that are positive by both swabs are plotted in Figure 1 as we aimed to evaluate the agreement of CT-values.

**2.10.** I found it interesting that the authors note that 43 participants opted out of providing paired samples, and 16 of those were unwilling after viewing the demonstration video. The Rhinoswab is marketed as a less invasive, patient-friendly alternative, but the video deterred some patients. No response for this comment is needed from the authors.

Thank you.

**Reviewer #3:** Reviewer
It is a pleasure to review the manuscript entitled "Agreement of Rhinoswabs and joint nose and throat swabs for detecting influenza and SARS-CoV-2 among symptomatic ambulatory patients in Hong Kong” (PONE-D-26-15719). I have carefully reviewed this manuscript. I found that it is straightforward, the sample size is reasonable (488 paired samples), the statistics are appropriate for the objective, and the conclusions are generally supported by the data. This manuscript appears scientifically sound, clinically relevant, and appropriate for publication in PLOS ONE. In particular, the manuscript is concise and well written. Nevertheless, a few minor issues should be addressed to strengthen the manuscript. After this revision, I support accepting this manuscript in PLOS ONE.

Thank you.

Manor’s suggestion for revision
**3.1**. The study mainly reports “concordance” but does not provide sensitivity, specificity, positive predictive agreement, negative predictive agreement, and Cohen’s kappa. Because NTS is effectively treated as a reference standard, reporting these metrics would strengthen diagnostic interpretation. Thus, the study would be strengthened by providing additional reporting of the sensitivity, specificity, positive/negative agreement, and Cohen’s kappa statistics to provide a more comprehensive assessment of diagnostic performance.

Thank you. We initially did not set out to treat NTS as the reference standard but have since added a supplementary table reporting the sensitivity, specificity and positive/negative agreement and subsequently added text to the results. Cohen’s kappa statistics have also been included in the results section of the main text.

**3.2**. The Rhinoswab was always collected AFTER rapid antigen testing and joint NTS sampling. This could reduce the amount of viral material remaining in the nose. The authors briefly mention this in the discussion. I think this deserves stronger acknowledgment. Because Rhinoswab collection was performed after other respiratory sampling procedures, the order of sampling may have influenced viral recovery. Please discuss this potential sampling-order bias in greater detail and clarify whether randomization of sampling order was considered.

Thank you. We have edited the third paragraph of the discussion to be more explicit on the sampling order, our findings, and also mentioning that we cannot evaluate the agreement between swabs had the Rhinoswab been sampled in a different order. We did not consider

randomizing the sampling order for this study.

**3.3** I found a very important issue. The manuscript promotes Rhinoswab as suitable for easy or minimally invasive collection, yet all samples were collected by trained staff. Thus, the conclusions about usability and self-testing are somewhat overstated. Because all Rhinoswab specimens were collected under supervision by trained staff, the manuscript should avoid overgeneralizing the findings to unsupervised self-collection settings.

Thank you. We have edited the discussion (lines 189 to 193) and conclusion (line 194) to avoid overstating and re-emphasizing the tests were carried out by trained professional. In addition, we call for further research in self-tested participants.

**3.4.** The authors used Ct ≤ 40 as the positivity cutoff. However, a Ct of 40 is relatively permissive. Please justify the use of a Ct of 40 as the positivity threshold and indicate whether equivocal results were observed near the cutoff.

Thank you. There were four samples that had a Ct-value ≥39. This relatively permissive threshold is particularly appropriate for the current study design. We have used data from an ongoing outpatient surveillance study. Enrolling participants within 3 days of symptom onset could result in detecting individuals early in infection when viral loads may still be rising and Ct values can be higher. Using a lower cutoff (e.g., <35) would risk missing true early or low-viral-load infections that are relevant for surveillance purposes.

**3.5**. The manuscript reports concordance percentages but only briefly presents confidence intervals. Please ensure that all concordance estimates include 95% confidence intervals in the main text and/or tables.

Thank you. This has been edited in text accordingly. All estimates include their 95% CI unless we mention the range of values (line 146).

**3.6.** Please correct minor typographical and formatting errors throughout the manuscript.

Thank you. These have been addressed in the figure and table legends and line 28 of the main text.

**Reviewer #4:** The manuscript addresses a practical question: whether a minimally invasive anterior nasal Rhinoswab can provide comparable RT-PCR detection of influenza A/B and SARS-CoV-2 to joint nose-and throat swabs in symptomatic outpatients. Overall, the study is clearly written, the sample size is reasonable, and the paired-sample design is appropriate for assessing diagnostic agreement. The reported concordance is high for all three viruses, and the analysis of Ct-value correlation and discordant samples supports the main conclusion that Rhinoswab may be a practical alternative in ambulatory settings.
Thank you.

**4.1.** However, the manuscript would benefit from a more careful framing of "agreement" versus diagnostic performance. Because neither specimen type is treated as an independent gold standard, term such as sensitivity or diagnostic accuracy should be avoided unless a composite reference standard is defined. The authors appropriately report concordance, but the Discussion occasionally implies broader diagnostic equivalence. This should be softened.
Thank you. We have subsequently changed some of the language used in the results and discussion (e.g. performance to agreement).

**4.2.** The statistical analysis is somewhat limited. Percent agreement alone maybe inflated by the large number of negative samples. The authors should consider adding positive percent agreement, negative percent agreement, and Cohen's kappa for each virus. Given the paired design, a McNemar test or equivalent comparison of discordant pairs would also strengthen the analysis. Confidence intervals should be provided consistently for all agreement metrics.
Thank you. We have now added Cohens kappa to the results main text and the sensitivity, specificity, positive percent agreement, negative percent agreement to a table in the supplementary materials.

**4.3.** The sequential sampling order is another important limitation. Rhinoswab was collected after rapid antigen testing and joint NTS, which may affect available nasal material. The authors interpret the preserved performance as reassuring, but fixed order still prevents assessments of whether Rhinoswab performance would be similar if collected first. This should be stated more explicitly.
Thank you. We have edited the third paragraph of the discussion and the last sentence now mentions the sampling order: “Nevertheless, the fixed sampling order precludes assessment of whether agreement would be equivalent (or potentially superior) if the Rhinoswab had been collected first”.

**4.4.** The manuscript also needs cleaner reporting of discordant results. A small table indicating which swab was positive, virus detected, Ct values, and symptom timing would help readers understand whether discordance reflects low viral load, sampling variability, or possible false positives.
Thank you. We have now added a supplementary table reporting the number of samples that were concordant or discordant between swabs.

**4.5.** Finally, the conclusion should be slightly narrower. The data support use in supervised collection among symptomatic outpatients early after symptom onset, but do not yet establish performance for self-collection, asymptomatic screening, later illness. or inpatient populations.
Thank you. We have edited the conclusion beginning at line 194 and additionally in the abstract.

**4.6.** Overall, this is a concise and useful study with practical implications. i would support publication after minor revision, mainly to improve statistical reporting, clarify limitations, and avoid overinterpreting concordance as full diagnostic equivalence.

Thank you.

**Reviewer #5:** Firstly, I truly appreciate for giving me this opportunity and selecting me to be apart of this work.

After carefully reviewing- Minor to moderate changes are required.
Clinically relevant study, highly suitable for publication.
Real time outpatient settings and patient population
Pairing of samples
Inclusion of three different respiratory viruses and large sample size study before conducting rhinoswab studies.

The topic is very interesting. Based on scientific reasoning- the conclusion is that ANS captures enough or sufficient viral RNA for reliable RT PCR in symptomatic patients is because respiratory viruses both SARS COV2 virus, RSV and influenza viruses-viral replication is more in the nasal cavity and helps in premature detection during infection and therefore, nasal swabs diagnostically works well during initial stages and thats why rhinoswabs are enough for molecular diagnostic detection of viruses.
Even MERS virus, other human coronovirus, human bocavirus, Adenoviruses, Human Parainfluenza Viruses, Human Metapneumovirus common rhinovirus can be detected-even though there is low viral load but still can be captured.

Therefore, ANS is a friendly, easier to use and delivers the same results.
A great tool for detection leading to diagnosis of the viruses.
It has the same quality and accuracy that helps in faster outcomes and optimizes health outcomes.
Cost efficient
Flexible
Validation of care

Concise and well executed diagnostic concordance study.
The concept and comparison is excellent.
The findings from the study is a feasible and alternative specimen specially in outpatient settings particularly given the high concordance observed.

Thank you for the general comments above.

**5.1.** Prior to publication-several methodological and reporting issues should be clarified.
The reporting is more general and overall agreement of percentages.
Additional reporting includes- Negative and positive percent agreement and cohen Kappa statistics. These metrics will help in method comparison.
The exact distribution in terms of samples is unclear- Only 18 paired samples were discordant- Positive by NTS only vs Rhinoswab only must be provided.
Virus specific Discordance counts
Provision of CT values for both specimen types
To strengthen the interpretation summarizing all the discordant specimens.

Thank you. We now report the Cohens kappa statistic in the results section of the main text. Negative percent agreement, positive percent agreement and the virus specific discordance counts have now been added as a supplementary table and mentioned further in text in the results section of the manuscript.

A second supplementary table also reports the number of concordant and discordant samples between swabs and their mean Ct-values.

**5.2.** Clarification of sampling order was fixed with participants, nostril sampling was repeatedly sampling.
Randomization order was considered

Thank you. We did not consider randomizing the order of sample collection. We have since edited the third paragraph of the discussion to state the sampling order more explicitly.

**5.3.** Since it is a self collection, the collection is lacking, self collection feasibility and evidence supporting diagnostic performance under supervised collection. The whole advantage of the rhinoswab is self collection and was collected by study staff.

Thank you. We have mentioned this as a limitation and possible future work.

**5.4.** There is a room for improvement on statistical reporting- Correlation alone does not assess concordance with agreements.
For the improvement use of Bland Altmann Analysis or report mean ct differences considering limitations.
Helps in systematic bias on specimens.
Thank you. We have now included Cohen’s kappa (with 95% confidence intervals) as a measure of agreement between Rhinoswab and joint NTS for each virus.

Regarding the Ct value analysis, in addition to Pearson correlation coefficients we included difference plots (Rhinoswab Ct minus NTS Ct versus NTS Ct) in Figure 1 (panels E–H). These plots are conceptually similar to a Bland-Altman analysis and allow visual assessment of variability in agreement across the range of viral loads

**5.5.** The title can be streamlined. Diagnostic concordance or agreement between Rhinoswab ans and jns for detection of influenza virus and SARS COV2.

Thank you. The title has been edited slightly to “Diagnostic agreement between Rhinoswabs and joint nose and throat swabs for the detection of influenza and SARS-CoV-2 among symptomatic ambulatory patients in Hong Kong.”

We have still included the population as it is important to highlight that it is among a symptomatic population.

**5.6**. Redundancy - Informed consent statement appears twice in the methods sections. – Thank you. We have removed the duplication of the written informed consent from the methods section.

**5.7.** CT value <40 (Clarification of positivity threshold format)

Thank you. This threshold is particularly appropriate for the current study design. We have used data from an ongoing outpatient surveillance study. Enrolling participants within 3 days of symptom onset could result in detecting individuals early in infection when viral loads may still be rising and Ct values can be higher. Using a lower cutoff (e.g., <35) would risk missing true early or low-viral-load infections that are relevant for surveillance purposes.

**5.8.** Typographical issues
formatting issues
spacing
figure labelling

Thank you. The figure 1 legend has been edited to be more descriptive and typographical errors and formatting issues have been addressed in the table and figure legends.

**5.9.** After interpretation - the overall study does not exactly show or talk about anterior nasal sampling captures higher viral loads that NTS.
It shows that ANS captures enough or sufficient viral RNA for reliable RT PCR in symptomatic patients and in outpatient settings.

Thank you. We agree that our findings demonstrate that anterior nasal sampling with the Rhinoswab captures sufficient viral RNA for reliable RT-qPCR detection in symptomatic outpatients, rather than systematically higher viral loads compared to joint NTS.
