# Supplementary material for: Diagnostic agreement between Rhinoswabs and joint nose and throat swabs for the detection of influenza and SARS-CoV-2 among symptomatic ambulatory patients in Hong Kong
Source: PLoS One. 2026 Aug 11;21(8):e0355869. doi: 10.1371/journal.pone.0355869 (PMC13460600; doi:10.1371/journal.pone.0355869)
Supplement: Peer review history

# Peer review: Decision Letter 0

**DOI:** 10.1371/journal.pone.0355869.r001

**Authors:** Hin Fung Tsang

3 Jun 2026

Dear Dr. Cowling,

Thank you for submitting your manuscript to PLOS ONE. After careful consideration, we feel that it has merit but does not fully meet PLOS ONE’s publication criteria as it currently stands. Therefore, we invite you to submit a revised version of the manuscript that addresses the points raised during the review process.

Please submit your revised manuscript by Jul 18 2026 11:59PM. If you will need more time than this to complete your revisions, please reply to this message or contact the journal office at plosone@plos.org. When you're ready to submit your revision, log on to https://www.editorialmanager.com/pone/ and select the 'Submissions Needing Revision' folder to locate your manuscript file.

- A letter that responds to each point raised by the academic editor and reviewer(s). You should upload this letter as a separate file labeled 'Response to Reviewers'.
- A marked-up copy of your manuscript that highlights changes made to the original version. You should upload this as a separate file labeled 'Revised Manuscript with Track Changes'.
- An unmarked version of your revised paper without tracked changes. You should upload this as a separate file labeled 'Manuscript'.

If you would like to make changes to your financial disclosure, please include your updated statement in your cover letter. Guidelines for resubmitting your figure files are available below the reviewer comments at the end of this letter.

If applicable, we recommend that you deposit your laboratory protocols in protocols.io to enhance the reproducibility of your results. Protocols.io assigns your protocol its own identifier (DOI) so that it can be cited independently in the future. For instructions see: https://journals.plos.org/plosone/s/submission-guidelines#loc-laboratory-protocols. Additionally, PLOS ONE offers an option for publishing peer-reviewed Lab Protocol articles, which describe protocols hosted on protocols.io. Read more information on sharing protocols at https://plos.org/protocols?utm_medium=editorial-email&utm_source=authorletters&utm_campaign=protocols.

As the corresponding author, your ORCID iD is verified in the submission system and will appear in the published article. PLOS supports the use of ORCID, and we encourage all coauthors to register for an ORCID iD and use it as well. Please encourage your coauthors to verify their ORCID iD within the submission system before final acceptance, as unverified ORCID iDs will not appear in the published article. *Only*  the individual author can complete the verification step; PLOS staff *cannot*  verify ORCID iDs on behalf of authors.

We look forward to receiving your revised manuscript.

Kind regards,

Hin Fung Tsang

Academic Editor

PLOS One

**Journal Requirements:**

1. When submitting your revision, we need you to address these additional requirements.

Please ensure that your manuscript meets PLOS ONE's style requirements, including those for file naming. The PLOS ONE style templates can be found at

https://journals.plos.org/plosone/s/file?id=wjVg/PLOSOne_formatting_sample_main_body.pdf and

https://journals.plos.org/plosone/s/file?id=ba62/PLOSOne_formatting_sample_title_authors_affiliations.pdf

2. Thank you for stating in your Funding Statement:

This research was financially supported by a grant from the Health and Medical Research Fund, Health Bureau, the Government of the Hong Kong Special Administrative Region (grant number INF-HKU-3) and the Theme-based Research Scheme (grant number T11-712/19-N) of the Research Grants Council of the Hong Kong SAR Government.

Please provide an amended statement that declares *all* the funding or sources of support (whether external or internal to your organization) received during this study, as detailed online in our guide for authors at http://journals.plos.org/plosone/s/submit-now.  Please also include the statement “There was no additional external funding received for this study.” in your updated Funding Statement.

Please include your amended Funding Statement within your cover letter. We will change the online submission form on your behalf.

3. Thank you for stating the following financial disclosure:

This research was financially supported by a grant from the Health and Medical Research Fund, Health Bureau, the Government of the Hong Kong Special Administrative Region (grant number INF-HKU-3) and the Theme-based Research Scheme (grant number T11-712/19-N) of the Research Grants Council of the Hong Kong SAR Government.

Please state what role the funders took in the study.  If the funders had no role, please state: "The funders had no role in study design, data collection and analysis, decision to publish, or preparation of the manuscript."

If this statement is not correct you must amend it as needed.

Please include this amended Role of Funder statement in your cover letter; we will change the online submission form on your behalf.

4. Thank you for stating the following in the Competing Interests section:

BJC consults for AstraZeneca, Fosun Pharma, GSK, Haleon, Moderna, Novavax, Pfizer, Roche, Sanofi Pasteur, and Seqirus. The authors report no other potential conflicts of interest.

We note that one or more of the authors are employed by a commercial company.

a. Please provide an amended Funding Statement declaring this commercial affiliation, as well as a statement regarding the Role of Funders in your study. If the funding organization did not play a role in the study design, data collection and analysis, decision to publish, or preparation of the manuscript and only provided financial support in the form of authors' salaries and/or research materials, please review your statements relating to the author contributions, and ensure you have specifically and accurately indicated the role(s) that these authors had in your study. You can update author roles in the Author Contributions section of the online submission form.

Please also include the following statement within your amended Funding Statement.

“The funder provided support in the form of salaries for authors [insert relevant initials], but did not have any additional role in the study design, data collection and analysis, decision to publish, or preparation of the manuscript. The specific roles of these authors are articulated in the ‘author contributions’ section.”

If your commercial affiliation did play a role in your study, please state and explain this role within your updated Funding Statement.

b. Please also provide an updated Competing Interests Statement declaring this commercial affiliation along with any other relevant declarations relating to employment, consultancy, patents, products in development, or marketed products, etc.

Within your Competing Interests Statement, please confirm that this commercial affiliation does not alter your adherence to all PLOS ONE policies on sharing data and materials by including the following statement: "This does not alter our adherence to  PLOS ONE policies on sharing data and materials.” (as detailed online in our guide for authors http://journals.plos.org/plosone/s/competing-interests) . If this adherence statement is not accurate and  there are restrictions on sharing of data and/or materials, please state these. Please note that we cannot proceed with consideration of your article until this information has been declared.

Please include both an updated Funding Statement and Competing Interests Statement in your cover letter. We will change the online submission form on your behalf.

5. Thank you for stating the following in the Competing Interests section:

BJC consults for AstraZeneca, Fosun Pharma, GSK, Haleon, Moderna, Novavax, Pfizer, Roche, Sanofi Pasteur, and Seqirus. The authors report no other potential conflicts of interest.

Please confirm that this does not alter your adherence to all PLOS ONE policies on sharing data and materials, by including the following statement: "This does not alter our adherence to  PLOS ONE policies on sharing data and materials.” (as detailed online in our guide for authors http://journals.plos.org/plosone/s/competing-interests).  If there are restrictions on sharing of data and/or materials, please state these. Please note that we cannot proceed with consideration of your article until this information has been declared.

Please include your updated Competing Interests statement in your cover letter; we will change the online submission form on your behalf.

6. In the online submission form, you indicated that your data will be submitted to a repository upon acceptance.  We strongly recommend all authors deposit their data before acceptance, as the process can be lengthy and hold up publication timelines. Please note that, though access restrictions are acceptable now, your entire minimal  dataset will need to be made freely accessible if your manuscript is accepted for publication. This policy applies to all data except where public deposition would breach compliance with the protocol approved by your research ethics board. If you are unable to adhere to our open data policy, please kindly revise your statement to explain your reasoning and we will seek the editor's input on an exemption.

7. We note that Figure S1 A in your submission contain copyrighted images. All PLOS content is published under the Creative Commons Attribution License (CC BY 4.0), which means that the manuscript, images, and Supporting Information files will be freely available online, and any third party is permitted to access, download, copy, distribute, and use these materials in any way, even commercially, with proper attribution. For more information, see our copyright guidelines: http://journals.plos.org/plosone/s/licenses-and-copyright.

We require you to either present written permission from the copyright holder to publish these figures specifically under the CC BY 4.0 license, or remove the figures from your submission:

a. You may seek permission from the original copyright holder of Figure S1 A to publish the content specifically under the CC BY 4.0 license.

We recommend that you contact the original copyright holder with the Content Permission Form (http://journals.plos.org/plosone/s/file?id=7c09/content-permission-form.pdf) and the following text:

“I request permission for the open-access journal PLOS ONE to publish XXX under the Creative Commons Attribution License (CCAL) CC BY 4.0 (http://creativecommons.org/licenses/by/4.0/). Please be aware that this license allows unrestricted use and distribution, even commercially, by third parties. Please reply and provide explicit written permission to publish XXX under a CC BY license and complete the attached form.”

Please upload the completed Content Permission Form or other proof of granted permissions as an "Other" file with your submission.

In the figure caption of the copyrighted figure, please include the following text: “Reprinted from [ref] under a CC BY license, with permission from [name of publisher], original copyright [original copyright year].”

b. If you are unable to obtain permission from the original copyright holder to publish these figures under the CC BY 4.0 license or if the copyright holder’s requirements are incompatible with the CC BY 4.0 license, please either i) remove the figure or ii) supply a replacement figure that complies with the CC BY 4.0 license. Please check copyright information on all replacement figures and update the figure caption with source information. If applicable, please specify in the figure caption text when a figure is similar but not identical to the original image and is therefore for illustrative purposes only.

8. We note that Figure S1 B includes an image of a patient in the study.

As per the PLOS ONE policy (http://journals.plos.org/plosone/s/submission-guidelines#loc-human-subjects-research) on papers that include identifying, or potentially identifying, information, the individual(s) or parent(s)/guardian(s) must be informed of the terms of the PLOS open-access (CC-BY) license and provide specific permission for publication of these details under the terms of this license. Please download the Consent Form for Publication in a PLOS Journal (http://journals.plos.org/plosone/s/file?id=8ce6/plos-consent-form-english.pdf). The signed consent form should not be submitted with the manuscript, but should be securely filed in the individual's case notes. Please amend the methods section and ethics statement of the manuscript to explicitly state that the patient/participant has provided consent for publication: “The individual in this manuscript has given written informed consent (as outlined in PLOS consent form) to publish these case details”.

If you are unable to obtain consent from the subject of the photograph, you will need to remove the figure and any other textual identifying information or case descriptions for this individual.

9. Please include captions for your Supporting Information files at the end of your manuscript, and update any in-text citations to match accordingly. Please see our Supporting Information guidelines for more information: http://journals.plos.org/plosone/s/supporting-information.

10. If the reviewer comments include a recommendation to cite specific previously published works, please review and evaluate these publications to determine whether they are relevant and should be cited. There is no requirement to cite these works unless the editor has indicated otherwise. 

11. Please review your reference list to ensure that it is complete and correct. If you have cited papers that have been retracted, please include the rationale for doing so in the manuscript text, or remove these references and replace them with relevant current references. Any changes to the reference list should be mentioned in the rebuttal letter that accompanies your revised manuscript. If you need to cite a retracted article, indicate the article’s retracted status in the References list and also include a citation and full reference for the retraction notice.

[Note: HTML markup is below. Please do not edit.]

Reviewers' comments:

**Comments to the Author**

1. Is the manuscript technically sound, and do the data support the conclusions?

Reviewer #1: Yes

Reviewer #2: Yes

Reviewer #3: Yes

Reviewer #4: Yes

Reviewer #5: Yes

**********

2. Has the statistical analysis been performed appropriately and rigorously?

Reviewer #1: Yes

Reviewer #2: Yes

Reviewer #3: Yes

Reviewer #4: No

Reviewer #5: Yes

**********

3. Have the authors made all data underlying the findings in their manuscript fully available?

The PLOS Data policy

Reviewer #1: Yes

Reviewer #2: Yes

Reviewer #3: Yes

Reviewer #4: Yes

Reviewer #5: Yes

**********

4. Is the manuscript presented in an intelligible fashion and written in standard English?

Reviewer #1: Yes

Reviewer #2: Yes

Reviewer #3: Yes

Reviewer #4: Yes

Reviewer #5: Yes

**********

**Reviewer #1:**  The manuscript addresses an important and clinically relevant topic regarding minimally invasive respiratory virus sampling methods.

The paired-sample design and use of RT-PCR strengthen the technical quality of the study.

The results demonstrate high concordance between Rhinoswabs and joint nose and throat swabs, supporting the potential utility of Rhinoswabs in outpatient settings.

The manuscript is generally well written and logically organized.

Statistical analysis is appropriate; however, inclusion of Cohen’s kappa statistics, sensitivity, specificity, and predictive values would provide a more comprehensive assessment of diagnostic agreement.

Additional discussion regarding applicability in asymptomatic individuals and unsupervised self-collection settings would strengthen the manuscript.

**Reviewer #2:**  The manuscript by Murphy et al addresses a practical question for clinical diagnostics. The authors provide a clear evaluation of a less invasive Rhinoswab swabbing method compared with standard practices. The manuscript is methodologically sound. The results are useful in clinical practice. I have several comments to improve the clarity and robustness before acceptance.

There were 18 discordant samples stated in Table 1. Please specify which swab yielded the positive result in these cases. It will be useful to know whether the Rhinoswab failed to detect viruses found by the NTS, or if the NTS missed viruses detected by the Rhinoswab. This also helps to address the next comment.

Negative cases are the majority here, which could skew the concordant value. For example, there are 32 positive SARS-CoV-2 cases, which means 456 negative cases. Let’s assume that Rhioswab couldn’t get any SARS-CoV-2 virus at all, thus it would only generate negative cases. In this scenario, the concordant value could still be as high as 456/488 = 93.4%. Please comment on this in the Discussion session.

1. Please consider using RT-qPCR since Ct values are obtained from qPCR.

2. “Written informed consent..” is stated twice at lines 64 and 67.

3. How is the Ct value determined? Baseline-Threshold or regression method?

4. Have any abnormal amplification curves been observed? If yes, how were the abnormal curves handled? This information should be included in the manuscript.

5. It will also be useful to detail the discordant cases (see above). Such as, how many have tested positive by Rhinoswab, but negative by joint NTS?

6. Figure 1 legend: Please use NTS for consistency. Please indicate that the r is Pearson’s correlation (I assume).

7. Figure 1: There are two linear regression lines, a solid and a dashed one, in the figure. What are they?

8. Figure 1: I assume in the figure only positive cases are plotted. Please make this clear in the legend.

9. Figure 1: Please consider indicating the discordant samples, maybe with a different color, to support the conclusion at line 114.

10. I found it interesting that the authors note that 43 participants opted out of providing paired samples, and 16 of those were unwilling after viewing the demonstration video. The Rhinoswab is marketed as a less invasive, patient-friendly alternative, but the video deterred some patients. No response for this comment is needed from the authors.

**Reviewer #3:**  Reviewer

It is a pleasure to review the manuscript entitled "Agreement of Rhinoswabs and joint nose and throat swabs for detecting influenza and SARS-CoV-2 among symptomatic ambulatory patients in Hong Kong” (PONE-D-26-15719). I have carefully reviewed this manuscript. I found that it is straightforward, the sample size is reasonable (488 paired samples), the statistics are appropriate for the objective, and the conclusions are generally supported by the data. This manuscript appears scientifically sound, clinically relevant, and appropriate for publication in PLOS ONE. In particular, the manuscript is concise and well written. Nevertheless, a few minor issues should be addressed to strengthen the manuscript. After this revision, I support accepting this manuscript in PLOS ONE.

Manor’s suggestion for revision

1. The study mainly reports “concordance” but does not provide sensitivity, specificity, positive predictive agreement, negative predictive agreement, and Cohen’s kappa. Because NTS is effectively treated as a reference standard, reporting these metrics would strengthen diagnostic interpretation. Thus, the study would be strengthened by providing additional reporting of the sensitivity, specificity, positive/negative agreement, and Cohen’s kappa statistics to provide a more comprehensive assessment of diagnostic performance.

2. The Rhinoswab was always collected AFTER rapid antigen testing and joint NTS sampling. This could reduce the amount of viral material remaining in the nose. The authors briefly mention this in the discussion. I think this deserves stronger acknowledgment. Because Rhinoswab collection was performed after other respiratory sampling procedures, the order of sampling may have influenced viral recovery. Please discuss this potential sampling-order bias in greater detail and clarify whether randomization of sampling order was considered.

3. I found a very important issue. The manuscript promotes Rhinoswab as suitable for easy or minimally invasive collection, yet all samples were collected by trained staff. Thus, the conclusions about usability and self-testing are somewhat overstated. Because all Rhinoswab specimens were collected under supervision by trained staff, the manuscript should avoid overgeneralizing the findings to unsupervised self-collection settings.

4. The authors used Ct ≤ 40 as the positivity cutoff. However, a Ct of 40 is relatively permissive. Please justify the use of a Ct of 40 as the positivity threshold and indicate whether equivocal results were observed near the cutoff.

5. The manuscript reports concordance percentages but only briefly presents confidence intervals. Please ensure that all concordance estimates include 95% confidence intervals in the main text and/or tables.

6. Please correct minor typographical and formatting errors throughout the manuscript.

**Reviewer #4:**  The manuscript addresses a practical question: whether a minimally invasive anterior nasal Rhinoswab can provide comparable RT-PCR detection of influenza A/B and SARS-CoV-2 to joint nose-and throat swabs in symptomatic outpatients. Overall, the study is clearly written, the sample size is reasonable, and the paired-sample design is appropriate for assessing diagnostic agreement. The reported concordance is high for all three viruses, and the analysis of Ct-value correlation and discordant samples supports the main conclusion that Rhinoswab may be a practical alternative in ambulatory settings.

However, the manuscript would benefit from a more careful framing of "agreement" versus diagnostic performance. Because neither specimen type is treated as an independent gold standard, term such as sensitivity or diagnostic accuracy should be avoided unless a composite reference standard is defined. The authors appropriately report concordance, but the Discussion occasionally implies broader diagnostic equivalence. This should be softened.

The statistical analysis is somewhat limited. Percent agreement alone maybe inflated by the large number of negative samples. The authors should consider adding positive percent agreement, negative percent agreement, and Cohen's kappa for each virus. Given the paired design, a McNemar test or equivalent comparison of discordant pairs would also strengthen the analysis. Confidence intervals should be provided consistently for all agreement metrics.

The sequential sampling order is another important limitation. Rhinoswab was collected after rapid antigen testing and joint NTS, which may affect available nasal material. The authors interpret the preserved performance as reassuring, but fixed order still prevents assessments of whether Rhinoswab performance would be similar if collected first. This should be stated more explicitly.

The manuscript also needs cleaner reporting of discordant results. A small table indicating which swab was positive, virus detected, Ct values, and symptom timing would help readers understand whether discordance reflects low viral load, sampling variability, or possible false positives.

Finally, the conclusion should be slightly narrower. The data support use in supervised collection among symptomatic outpatients early after symptom onset, but do not yet establish performance for self-collection, asymptomatic screening, later illness. or inpatient populations.

Overall, this is a concise and useful study with practical implications. i would support publication after minor revision, mainly to improve statistical reporting, clarify limitations, and avoid overinterpreting concordance as full diagnostic equivalence.

**Reviewer #5:**  Firstly, I truly appreciate for giving me this opportunity and selecting me to be apart of this work.

After carefully reviewing- Minor to moderate changes are required.

Clinically relevant study, highly suitable for publication.

Real time outpatient settings and patient population

Pairing of samples

Inclusion of three different respiratory viruses and large sample size study before conducting rhinoswab studies.

The topic is very interesting. Based on scientific reasoning- the conclusion is that ANS captures enough or sufficient viral RNA for reliable RT PCR in symptomatic patients is because respiratory viruses both SARS COV2 virus, RSV and influenza viruses-viral replication is more in the nasal cavity and helps in premature detection during infection and therefore, nasal swabs diagnostically works well during initial stages and thats why rhinoswabs are enough for molecular diagnostic detection of viruses.

Even MERS virus, other human coronovirus, human bocavirus, Adenoviruses, Human Parainfluenza Viruses, Human Metapneumovirus common rhinovirus can be detected-even though there is low viral load but still can be captured.

There fore, ANS is a friendly, easier to use and delivers the same results.

A great tool for detection leading to diagnosis of the viruses.

It has the same quality and accuracy that helps in faster outcomes and optimizes health outcomes.

Cost efficient

Flexible

Validation of care

Concise and well executed diagnostic concordance study.

The concept and comparison is excellent.

The findings from the study is a feasible and alternative specimen specially in outpatient settings particularly given the high concordance observed.

Prior to publication-several methodological and reporting issues should be clarified.

The reporting is more general and overall agreement of percentages.

Additional reporting includes- Negative and positive percent agreement and cohen Kappa statistics. These metrics will help in method comparison.

The exact distribution in terms of samples is unclear- Only 18 paired samples were discordant- Positive by NTS only vs Rhinoswab only must be provided.

Virus specific Discordance counts

Provision of CT values for both specimen types

To strengthen the interpretation summarizing all the discordant specimens.

Clarification of sampling order was fixed with participants, nostril sampling was repeatedly sampling.

Randomization order was considered

Since it is a self collection, the collection is lacking, self collection feasibility and evidence supporting diagnostic performance under supervised collection. The whole advantage of the rhinoswab is self collection and was collected by study staff.

There is a room for improvement on statistical reporting- Correlation alone does not assess concordance with agreements.

For the improvement use of Bland Altmann Analysis or report mean ct differences considering limitations.

Helps in systematic bias on specimens.

The title can be streamlined. Diagnostic concordance or agreement between Rhinoswab ans and jns for detection of influenza virus and SARS COV2.

Redundancy - Informed consent statement appears twice in the methods sections.

CT value <40 (Clarification of positivity threshold format)

Typographical issues

formatting issues

spacing

figure labelling

After interpretation - the overall study does not exactly show or talk about anterior nasal sampling captures higher viral loads that NTS.

It shows that ANS captures enough or sufficient viral RNA for reliable RT PCR in symptomatic patients and in outpatient settings.

**********

what does this mean?). If published, this will include your full peer review and any attached files.

If you choose “no”, your identity will remain anonymous but your review may still be made public.

**Do you want your identity to be public for this peer review?**  For information about this choice, including consent withdrawal, please see our Privacy Policy

Reviewer #1: **Yes:**  DR LAKSHMI JYOTHI T

Reviewer #2: No

Reviewer #3: No

Reviewer #4: No

Reviewer #5: **Yes:**  MONISHA KANDALA

**********

[NOTE: If reviewer comments were submitted as an attachment file, they will be attached to this email and accessible via the submission site. Please log into your account, locate the manuscript record, and check for the action link "View Attachments". If this link does not appear, there are no attachment files.]

To ensure your figures meet our technical requirements, please review our figure guidelines: https://journals.plos.org/plosone/s/figures

You may also use PLOS’s free figure tool, NAAS, to help you prepare publication quality figures: https://journals.plos.org/plosone/s/figures#loc-tools-for-figure-preparation.

NAAS will assess whether your figures meet our technical requirements by comparing each figure against our figure specifications.

Attachment Submitted filename: PONE-D-26-15719-2.pdf

Attachment Submitted filename: Reviewer 1 recommendation.pdf

# Author comment: Author response to Decision Letter 1

**DOI:** 10.1371/journal.pone.0355869.r002

23 Jul 2026

Journal Requirements:

1. When submitting your revision, we need you to address these additional requirements.

Please ensure that your manuscript meets PLOS ONE's style requirements, including those for file naming. The PLOS ONE style templates can be found at

https://journals.plos.org/plosone/s/file?id=wjVg/PLOSOne_formatting_sample_main_body.pdf and

https://journals.plos.org/plosone/s/file?id=ba62/PLOSOne_formatting_sample_title_authors_affiliations.pdf

Thank you. The manuscript has been edited accordingly.

2. Thank you for stating in your Funding Statement:

This research was financially supported by a grant from the Health and Medical Research Fund, Health Bureau, the Government of the Hong Kong Special Administrative Region (grant number INF-HKU-3) and the Theme-based Research Scheme (grant number T11-712/19-N) of the Research Grants Council of the Hong Kong SAR Government.

Please provide an amended statement that declares *all* the funding or sources of support (whether external or internal to your organization) received during this study, as detailed online in our guide for authors at http://journals.plos.org/plosone/s/submit-now. Please also include the statement “There was no additional external funding received for this study.” in your updated Funding Statement.

Please include your amended Funding Statement within your cover letter. We will change the online submission form on your behalf.

We have updated the statement to include that there was no additional funding received for this study.

This research was financially supported by a grant received by BJC from the Health and Medical Research Fund, Health Bureau, the Government of the Hong Kong Special Administrative Region (grant number INF-HKU-3) and the Theme-based Research Scheme (grant number T11-712/19-N) of the Research Grants Council of the Hong Kong SAR Government. There was no additional external funding received for this study. The funders had no role in study design, data collection and analysis, decision to publish, or preparation of the manuscript.

3. Thank you for stating the following financial disclosure:

This research was financially supported by a grant from the Health and Medical Research Fund, Health Bureau, the Government of the Hong Kong Special Administrative Region (grant number INF-HKU-3) and the Theme-based Research Scheme (grant number T11-712/19-N) of the Research Grants Council of the Hong Kong SAR Government.

Please state what role the funders took in the study. If the funders had no role, please state: "The funders had no role in study design, data collection and analysis, decision to publish, or preparation of the manuscript."

If this statement is not correct you must amend it as needed.

Please include this amended Role of Funder statement in your cover letter; we will change the online submission form on your behalf.

The funders had no role and therefore the above statement has been included in the manuscript.

4. Thank you for stating the following in the Competing Interests section:

BJC consults for AstraZeneca, Fosun Pharma, GSK, Haleon, Moderna, Novavax, Pfizer, Roche, Sanofi Pasteur, and Seqirus. The authors report no other potential conflicts of interest.

We note that one or more of the authors are employed by a commercial company.

a. Please provide an amended Funding Statement declaring this commercial affiliation, as well as a statement regarding the Role of Funders in your study. If the funding organization did not play a role in the study design, data collection and analysis, decision to publish, or preparation of the manuscript and only provided financial support in the form of authors' salaries and/or research materials, please review your statements relating to the author contributions, and ensure you have specifically and accurately indicated the role(s) that these authors had in your study. You can update author roles in the Author Contributions section of the online submission form.

Please also include the following statement within your amended Funding Statement.

“The funder provided support in the form of salaries for authors [insert relevant initials], but did not have any additional role in the study design, data collection and analysis, decision to publish, or preparation of the manuscript. The specific roles of these authors are articulated in the ‘author contributions’ section.”

If your commercial affiliation did play a role in your study, please state and explain this role within your updated Funding Statement.

The Centre for Immunology and Infection provided support in the form of salaries for authors (MP) but did not have any additional role in the study design, data collection and analysis, decision to publish, or preparation of the manuscript. The specific roles of these authors are articulated in the ‘author contributions’ section.

b. Please also provide an updated Competing Interests Statement declaring this commercial affiliation along with any other relevant declarations relating to employment, consultancy, patents, products in development, or marketed products, etc.

Within your Competing Interests Statement, please confirm that this commercial affiliation does not alter your adherence to all PLOS ONE policies on sharing data and materials by including the following statement: "This does not alter our adherence to PLOS ONE policies on sharing data and materials.” (as detailed online in our guide for authors http://journals.plos.org/plosone/s/competing-interests) . If this adherence statement is not accurate and there are restrictions on sharing of data and/or materials, please state these. Please note that we cannot proceed with consideration of your article until this information has been declared.

Please include both an updated Funding Statement and Competing Interests Statement in your cover letter. We will change the online submission form on your behalf.

There is no relevant commercial interest for C2I. This does not alter our adherence to PLOS ONE policies on sharing data and materials.

5. Thank you for stating the following in the Competing Interests section:

BJC consults for AstraZeneca, Fosun Pharma, GSK, Haleon, Moderna, Novavax, Pfizer, Roche, Sanofi Pasteur, and Seqirus. The authors report no other potential conflicts of interest.

Please confirm that this does not alter your adherence to all PLOS ONE policies on sharing data and materials, by including the following statement: "This does not alter our adherence to PLOS ONE policies on sharing data and materials.” (as detailed online in our guide for authors http://journals.plos.org/plosone/s/competing-interests). If there are restrictions on sharing of data and/or materials, please state these. Please note that we cannot proceed with consideration of your article until this information has been declared.

Please include your updated Competing Interests statement in your cover letter; we will change the online submission form on your behalf.

Thank you. We have added the statement: “This does not alter our adherence to PLOS ONE policies on sharing data and materials”. The data has also now been made available on GitHub.

6. In the online submission form, you indicated that your data will be submitted to a repository upon acceptance. We strongly recommend all authors deposit their data before acceptance, as the process can be lengthy and hold up publication timelines. Please note that, though access restrictions are acceptable now, your entire minimal dataset will need to be made freely accessible if your manuscript is accepted for publication. This policy applies to all data except where public deposition would breach compliance with the protocol approved by your research ethics board. If you are unable to adhere to our open data policy, please kindly revise your statement to explain your reasoning and we will seek the editor's input on an exemption.

Thank you. The data have also now been made available on GitHub.

7. We note that Figure S1 A in your submission contain copyrighted images. All PLOS content is published under the Creative Commons Attribution License (CC BY 4.0), which means that the manuscript, images, and Supporting Information files will be freely available online, and any third party is permitted to access, download, copy, distribute, and use these materials in any way, even commercially, with proper attribution. For more information, see our copyright guidelines: http://journals.plos.org/plosone/s/licenses-and-copyright.

We require you to either present written permission from the copyright holder to publish these figures specifically under the CC BY 4.0 license, or remove the figures from your submission:

a. You may seek permission from the original copyright holder of Figure S1 A to publish the content specifically under the CC BY 4.0 license.

We recommend that you contact the original copyright holder with the Content Permission Form (http://journals.plos.org/plosone/s/file?id=7c09/content-permission-form.pdf) and the following text:

“I request permission for the open-access journal PLOS ONE to publish XXX under the Creative Commons Attribution License (CCAL) CC BY 4.0 (http://creativecommons.org/licenses/by/4.0/). Please be aware that this license allows unrestricted use and distribution, even commercially, by third parties. Please reply and provide explicit written permission to publish XXX under a CC BY license and complete the attached form.”

Please upload the completed Content Permission Form or other proof of granted permissions as an "Other" file with your submission.

In the figure caption of the copyrighted figure, please include the following text: “Reprinted from [ref] under a CC BY license, with permission from [name of publisher], original copyright [original copyright year].”

For Figure S1 A, we used our own stock of swabs and took the picture ourselves and this picture is not copyrighted.

b. If you are unable to obtain permission from the original copyright holder to publish these figures under the CC BY 4.0 license or if the copyright holder’s requirements are incompatible with the CC BY 4.0 license, please either i) remove the figure or ii) supply a replacement figure that complies with the CC BY 4.0 license. Please check copyright information on all replacement figures and update the figure caption with source information. If applicable, please specify in the figure caption text when a figure is similar but not identical to the original image and is therefore for illustrative purposes only.

8. We note that Figure S1 B includes an image of a patient in the study.

As per the PLOS ONE policy (http://journals.plos.org/plosone/s/submission-guidelines#loc-human-subjects-research) on papers that include identifying, or potentially identifying, information, the individual(s) or parent(s)/guardian(s) must be informed of the terms of the PLOS open-access (CC-BY) license and provide specific permission for publication of these details under the terms of this license. Please download the Consent Form for Publication in a PLOS Journal (http://journals.plos.org/plosone/s/file?id=8ce6/plos-consent-form-english.pdf). The signed consent form should not be submitted with the manuscript, but should be securely filed in the individual's case notes. Please amend the methods section and ethics statement of the manuscript to explicitly state that the patient/participant has provided consent for publication: “The individual in this manuscript has given written informed consent (as outlined in PLOS consent form) to publish these case details”.

If you are unable to obtain consent from the subject of the photograph, you will need to remove the figure and any other textual identifying information or case descriptions for this individual.

Thank you. The picture is of the manuscripts first author, not a study participant. We have consent and as such have updated the ethics statement of the manuscript.

The ethics statement now reads:

“The study protocol was approved by the Institutional Review Board of the University of Hong Kong. Written informed consent was obtained from all participants or their legal guardians for minors. The individual pictured in this manuscript has given written informed consent to publish the image.”

9. Please include captions for your Supporting Information files at the end of your manuscript, and update any in-text citations to match accordingly. Please see our Supporting Information guidelines for more information: http://journals.plos.org/plosone/s/supporting-information.

The Supporting Information section has now been added before the acknowledgements section of the paper, listing the captions of the supplementary figures and tables as well as linking the GitHub dataset and script.

https://github.com/caitmurphy/rhinoswab_concordance

10. If the reviewer comments include a recommendation to cite specific previously published works, please review and evaluate these publications to determine whether they are relevant and should be cited. There is no requirement to cite these works unless the editor has indicated otherwise.

Thank you. The reviewers did not indicate previously published works to reference.

11. Please review your reference list to ensure that it is complete and correct. If you have cited papers that have been retracted, please include the rationale for doing so in the manuscript text, or remove these references and replace them with relevant current references. Any changes to the reference list should be mentioned in the rebuttal letter that accompanies your revised manuscript. If you need to cite a retracted article, indicate the article’s retracted status in the References list and also include a citation and full reference for the retraction notice.

Thank you. The references have been edited to ensure all of them contain their doi as requested. We have not referenced any retracted papers or preprints.

Reviewer #1:

The manuscript addresses an important and clinically relevant topic regarding minimally invasive respiratory virus sampling methods.

The paired-sample design and use of RT-PCR strengthen the technical quality of the study.

The results demonstrate high concordance between Rhinoswabs and joint nose and throat swabs, supporting the potential utility of Rhinoswabs in outpatient settings.

The manuscript is generally well written and logically organized.

Thank you.

1.1 Statistical analysis is appropriate; however, inclusion of Cohen’s kappa statistics, sensitivity, specificity, and predictive values would provide a more comprehensive assessment of diagnostic agreement.

Thank you. We have added Cohen’s kappa statistics to the main text and a supplementary table containing the other diagnostic performance metrics.

1.2 Additional discussion regarding applicability in asymptomatic individuals and unsupervised self-collection settings would strengthen the manuscript.

Thank you. We have now mentioned that the concordance observed in our study may not generalize to those with lower viral loads or asymptomatic individuals and included further discussion on collection methods in the limitations paragraph of the discussion.

Reviewer #2: The manuscript by Murphy et al addresses a practical question for clinical diagnostics. The authors provide a clear evaluation of a less invasive Rhinoswab swabbing method compared with standard practices. The manuscript is methodologically sound. The results are useful in clinical practice. I have several comments to improve the clarity and robustness before acceptance.

Thank you.

There were 18 discordant samples stated in Table 1. Please specify which swab yielded the positive result in these cases. It will be useful to know whether the Rhinoswab failed to detect viruses found by the NTS, or if the NTS missed viruses detected by the Rhinoswab. This also helps to address the next comment.

Negative cases are the majority here, which could skew the concordant value. For example, there are 32 positive SARS-CoV-2 cases, which means 456 negative cases. Let’s assume that Rhioswab couldn’t get any SARS-CoV-2 virus at all, thus it would only generate negative cases. In this scenario,

Attachment Submitted filename: Response to Reviewers.docx

# Editor report: Decision Letter 1

**DOI:** 10.1371/journal.pone.0355869.r003

**Authors:** Hin Fung Tsang

27 Jul 2026

Diagnostic agreement between Rhinoswabs and joint nose and throat swabs for the detection of influenza and SARS-CoV-2 among symptomatic ambulatory patients in Hong Kong

PONE-D-26-15719R1

Dear Prof. Cowling,

We’re pleased to inform you that your manuscript has been judged scientifically suitable for publication and will be formally accepted for publication once it meets all outstanding technical requirements.

Within one week, you’ll receive an e-mail detailing the required amendments. When these have been addressed, you’ll receive a formal acceptance letter and your manuscript will be scheduled for publication.

An invoice will be generated when your article is formally accepted. Please note, if your institution has a publishing partnership with PLOS and your article meets the relevant criteria, all or part of your publication costs will be covered. Please make sure your user information is up-to-date by logging into Editorial Manager at Editorial Manager® and clicking the ‘Update My Information' link at the top of the page. For questions related to billing, please contact billing support.

If your institution or institutions have a press office, please notify them about your upcoming paper to help maximize its impact. If they’ll be preparing press materials, please inform our press team as soon as possible -- no later than 48 hours after receiving the formal acceptance. Your manuscript will remain under strict press embargo until 2 pm Eastern Time on the date of publication. For more information, please contact onepress@plos.org.

Kind regards,

Hin Fung Tsang

Academic Editor

PLOS One

Additional Editor Comments (optional):

Reviewers' comments:

# Editor report: Acceptance letter

**DOI:** 10.1371/journal.pone.0355869.r004

**Authors:** Hin Fung Tsang

PONE-D-26-15719R1

PLOS One

Dear Dr. Cowling,

I'm pleased to inform you that your manuscript has been deemed suitable for publication in PLOS One. Congratulations! Your manuscript is now being handed over to our production team.

At this stage, our production department will prepare your paper for publication. This includes ensuring the following:

* All references, tables, and figures are properly cited

* All relevant supporting information is included in the manuscript submission,

* There are no issues that prevent the paper from being properly typeset

You will receive further instructions from the production team, including instructions on how to review your proof when it is ready. Please keep in mind that we are working through a large volume of accepted articles, so please give us a few days to review your paper and let you know the next and final steps.

Lastly, if your institution or institutions have a press office, please let them know about your upcoming paper now to help maximize its impact. If they'll be preparing press materials, please inform our press team within the next 48 hours. Your manuscript will remain under strict press embargo until 2 pm Eastern Time on the date of publication. For more information, please contact onepress@plos.org.

You will receive an invoice from PLOS for your publication fee after your manuscript has reached the completed accept phase. If you receive an email requesting payment before acceptance or for any other service, this may be a phishing scheme. Learn how to identify phishing emails and protect your accounts at https://explore.plos.org/phishing.

If we can help with anything else, please email us at customercare@plos.org.

Thank you for submitting your work to PLOS ONE and supporting open access.

Kind regards,

PLOS ONE Editorial Office Staff

on behalf of

Dr. Hin Fung Tsang

Academic Editor

PLOS One
